# Development of PBPK Population Model for End-Stage Renal Disease Patients to Inform OATP1B-, BCRP-, P-gp-, and CYP3A4-Mediated Drug Disposition with Individual Influencing Factors

**DOI:** 10.3390/pharmaceutics17081078

**Published:** 2025-08-20

**Authors:** Yujie Wu, Weijie Kong, Jiayu Li, Xiaoqiang Xiang, Hao Liang, Dongyang Liu

**Affiliations:** 1 Department of Nephrology, Peking University Third Hospital, Beijing 100191, China; danielawu@yeah.net (Y.W.); kongweijie0712@foxmail.com (W.K.); rubyli0224@163.com (J.L.); 2 Drug Clinical Trial Center, Peking University Third Hospital, Beijing 100191, China; 3 Department of Clinical Pharmacy and Pharmacy Administration, School of Pharmacy, Fudan University, Shanghai 201203, China; xiangxq@fudan.edu.cn; 4 Institute of Medical Innovation, Peking University Third Hospital, Beijing 100191, China

**Keywords:** end-stage renal disease, drug-metabolizing enzyme and transporter, pharmacokinetics, physiologically based pharmacokinetic modeling, drug–drug–disease interaction

## Abstract

**Background/Objective:** Physiologically based pharmacokinetic (PBPK) modeling is a powerful tool for predicting pharmacokinetics (PK) to support drug development and precision medicine. However, it has not been established for non-renal clearance pathways in patients with end-stage renal disease (ESRD), a population that bears heavy medication burden and is thereby at high risk for drug–drug–disease interactions (DDDIs). Furthermore, the pronounced inter-individual variability in PK observed in ESRD patients highlights the urgent need for individualized PBPK models. **Methods:** In this study, we developed a PBPK population model for ESRD patients, incorporating functional changes in key drug-metabolizing enzymes and transporters (DMETs), including CYP3A4, OATP1B1/3, P-gp, and BCRP. The model was initially constructed using the recalibrated demographic and physiological parameters of ESRD patients. Then, we used five well-validated substrates (midazolam, dabigatran etexilate, pitavastatin, rosuvastatin, and atorvastatin) and their corresponding PK profiles from ESRD patients taking a microdose cocktail regimen to simultaneously estimate the abundance of all these DMETs. Lastly, machine learning was employed to identify potential factors influencing individual clearance. **Results:** Our study suggested a significant reduction in hepatic OATP1B1/3 (75%) and intestinal P-gp abundance (34%) in ESRD patients. Ileum BCRP abundance was estimated to increase by 100%, while change in hepatic CYP3A4 abundance is minimal. Notably, simulations of drug combinations revealed potential DDDI risks that were not observed in healthy volunteers. Machine learning further identified Clostridium XVIII and Escherichia genus abundances as significant factors influencing dabigatran clearance. For rosuvastatin, aspartate aminotransferase, total bilirubin, *Bacteroides*, and *Megamonas* genus abundances were key influencers. No significant factors were identified for midazolam, pitavastatin, or atorvastatin. **Conclusions:** Our study proposes a feasible strategy for individualized PK prediction by integrating PBPK modeling with machine learning to support the development and precise use of the aforementioned DMET substrates in ESRD patients.

## 1. Introduction

Chronic kidney disease (CKD) is a widespread global health issue with an estimated prevalence of 8–16% worldwide [1]. End-stage renal disease (ESRD) is the most severe and irreversible stage of CKD (glomerular filtration rate [GFR] < 15 mL/min/1.73 m^2^) and relies on dialysis. ESRD patients suffer from high risk of adverse drug reactions (ADRs) [2,3], which may be attributed to increased drug exposure resulting from three aspects. Firstly, in addition to reduced renal elimination, growing evidence indicates that non-renal drug-metabolizing enzyme and transporter (DMET) functions are also altered due to the accumulation of uremic toxins [4]. Consequently, the exposure of both renally and non-renally eliminated drugs in ESRD patients may change significantly [5,6,7]. Secondly, ESRD patients are often accompanied with multiple comorbidities, necessitating complicated medication regimens [8]. Polypharmacy is frequently associated with substantial drug–drug–disease interactions (DDDIs) and further exacerbates ADR risk [2,9,10]. Lastly, patients with advanced CKD exhibited greater inter-individual variability (IIV) in the pharmacokinetic (PK) profiles [11], potentially leading to unexpected ADR occurrences. Collectively, these challenges pose a significant barrier to the precision medicine in ESRD patients and solutions are urgently warranted.

Physiologically based pharmacokinetic (PBPK) modeling is a powerful tool to incorporate the synergistic impact of demographic, physiological, and genotypical parameters, as well as DMET functions [12]. As a result, PBPK models have been widely used in drug development and precision medicine in CKD patients by providing valuable insights for clinical trial design and dosage optimization [13]. PBPK population models have been established for patients with mild, moderate, and severe CKD [14]. Although these models could accurately predict drug exposures across different CKD stages, the predictive accuracy tends to decline as CKD severity increases [15]. Moreover, PK profiles in ESRD patients cannot be extrapolated from a severe CKD PBPK model, due to differences in physiological parameters and dialysis-mediated elimination of uremic toxins, which may impact DMET functions [16]. For instance, when using a severe CKD PBPK model to predict PK profiles of CYP2D6 substrate drugs in ESRD patients, only 20% of predictions fell within 2-fold of the observed area under the curve (AUC) [17]. Lastly, traditional PBPK models predict drug PK at the population level and are insufficient to handle the large IIVs in ESRD patients. Efforts have been made to establish individualized PBPK models to characterize the effect of hemodialysis on non-renal pathway functions in ESRD patients [18]. However, these models have not yet incorporated the impact of genotype [19] and gut microbiome [20], both of which profoundly contribute to the IIV of PK profiles in CKD patients. Collectively, the predictive performance of established PBPK models is unsatisfactory for PK prediction of ESRD patients and lacks considerations at the individual level, necessitating the development of a novel PBPK modeling approach.

P-glycoprotein (P-gp), breast cancer resistance protein (BCRP), and organic anion-transporting polypeptide 1 and 3 (OATP1B1/3) are the most contributive efflux or uptake transporters for drug disposition across multiple organs, while CYP3A4 plays a major role in the elimination of 64% of drugs newly approved by the US Food and Drug Administration (FDA) between 2005 and 2016 [21]. Midazolam (MDZ) is mainly metabolized by CYP3A4 [22]. Dabigatran etexilate (DABE) is a prodrug rapidly converted to its active moiety dabigatran (DAB) via two primary intermediate metabolites by carboxylesterase (CES)-2 in the intestine and CES-1/CES-2 in the liver. DABE (but not DAB) is a substrate of the efflux transporter P-gp [23]. Pitavastatin (PTV) is a sensitive and selective clinical probe substrate for OATP1B, undergoing minimal hepatic metabolism primarily via glucuronidation to form pitavastatin lactone [24]. Rosuvastatin (RSV) is predominantly eliminated through influx (OATP1B1/3) and efflux (BCRP) drug transporters, with enzymes providing only a minor contribution to its clearance [25]. Atorvastatin (ATV) is primarily eliminated through metabolic pathways (CYP3A4), active hepatic uptake byOATP1B1/3, and efflux by BCRP and P-gp [26].

In our previous study, PK profiles of MDZ, DAB, PTV, RSV, and ATV were simultaneously obtained in Chinese healthy volunteers (HVs) and ESRD patients following single-dose microdose cocktail administration [27]. The functional changes of these DMETs were preliminarily estimated by the population pharmacokinetic (PPK) models of the corresponding substrates. However, the translation of substrate exposure to non-renal DMET function is not straightforward due to the accompanied physiological alterations in CKD patients [28].

In this study, we aim to develop a novel PBPK model for ESRD patients that integrates physiological parameters and the functional changes of CYP3A4, OATP1B1/3, BCRP, and P-gp. The model will be used to (1) evaluate the impact of ESRD on substrates exposure when drugs are co-administered with perpetrators, thereby revealing potential DDDI risks in ESRD patients and (2) explore significant influencing factors for individual drug clearance.

## 2. Methods

### 2.1. Overall Strategy

**Figure 1:**
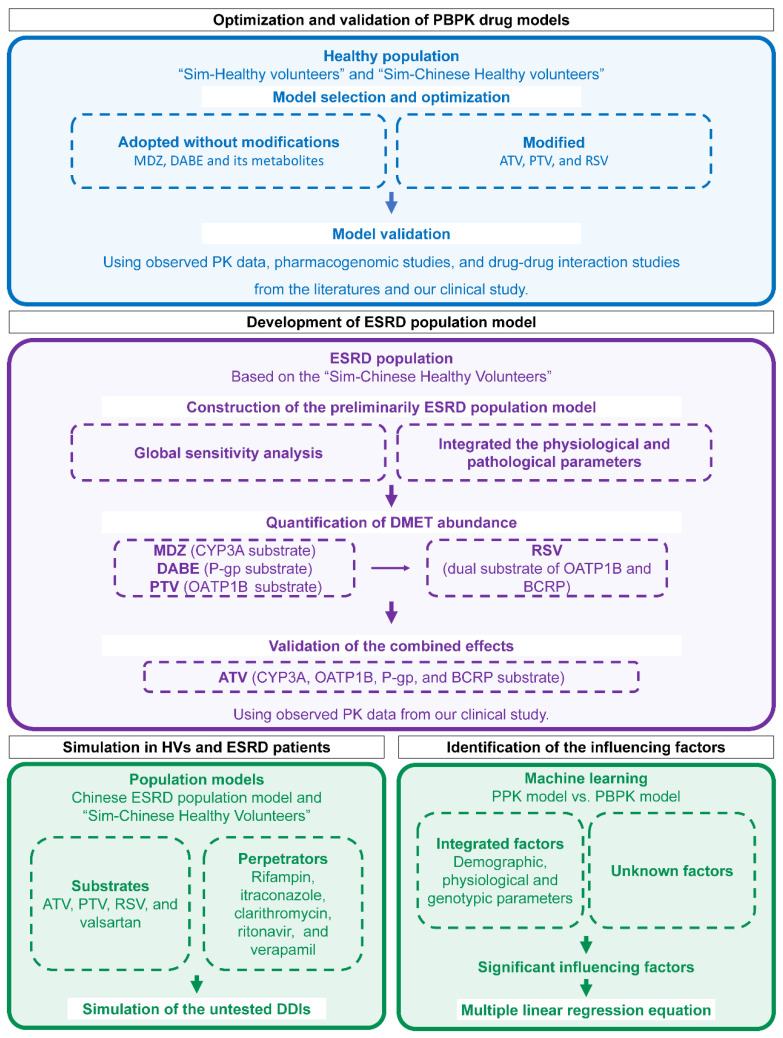
Workflow for PBPK modeling, simulation, and influencing-factors identification. PBPK physiologically based pharmacokinetic; MDZ midazolam; DABE, DAB, and DABG dabigatran etexilate and its metabolites dabigatran and dabigatran glucuronide; ATV atorvastatin; PTV pitavastatin; RSV rosuvastatin; PK pharmacokinetic; ESRD end-stage renal disease; DMET drug-metabolizing enzymes and transporter; HV healthy volunteers; DDI drug–drug interaction; PPK population pharmacokinetic.

The overall workflow is illustrated in Figure 1. Firstly, five substrate drug models were validated using PK data in HVs from the literature and our previous study [27]. Next, based on the built-in “Sim-Chinese Healthy Volunteers” population model in Simcyp, the ESRD population model was developed by incorporating physiological parameters and DMET functions specific to Chinese ESRD patients. Then, DDDIs between substrates and perpetrators were evaluated through simulations using the established model. Lastly, influencing factors for individual clearance were identified and quantified.

### 2.2. Refinement of PBPK Drug Models

The PBPK drug model for MDZ was obtained from the Simcyp compound library without modifications. The DABE drug model, along with its metabolites DAB and dabigatran glucuronide acid (DABG), had been previously developed and validated [29]. The PTV model was initially adopted from published literature [30], with the OATP1B1 and OATP1B3 active uptake scaling factors optimized to 14 based on pharmacogenomic (PGx) studies in Caucasian HVs (Figure S1) [31]. The built-in ATV and RSV models in Simcyp were refined to according to their respective drug disposition characteristics. Since ATV is a substrate of BCRP, BCRP-mediated efflux was incorporated into the model with an intrinsic clearance (CL_int,T_) value of 6 μL/min [26]. For RSV, renal clearance accounts for approximately 28% of its total plasma clearance [32]. Organic anion transporter 3 (OAT3) serves as the rate-limiting step in RSV renal clearance, while BCRP also contributes to its renal efflux. Consequently, renal OAT3- and BCRP-mediated efflux were included in the RSV model, with CL_int,T_ values assigned to 150 μL/min/million cells [33]. The parameters for all five drugs are summarized in Tables S1–S6.

### 2.3. Collection of Pharmacokinetic Data

Relevant clinical PK data for all five drugs were systematically searched in PubMed (https://www.ncbi.nlm.nih.gov/pubmed/) and Embase (https://www.embase.com) (accessed on 1 July 2023), using the keywords “healthy volunteer” and “pharmacokinetics”.

Inclusion criteria were as follows: (1) studies involving Caucasian or Chinese HVs; (2) age ≥ 18 years; (3) sample size ≥ 6; (4) availability of PK parameters or concentration–time profiles. Exclusion criteria included (1) use of sustained-release formulations or fixed-dose combination drugs; (2) studies with <50% Caucasian or Chinese participants; (3) statin samples without the addition of a stabilizing agent during detection.

Observed AUC and maximum concentration (C_max_) were collected from the literature. Plasma concentration–time curves were extracted using Graph Digitizer (Version 2.0).

In addition, PK data were obtained from our previous clinical study [27]. A total of 14 Chinese HVs and 10 ESRD patients were enrolled and received a single dose of microdose cocktail regimen containing 10 μg MDZ, 375 μg DABE, 10 μg PTV, 50 μg RSV, and 100 μg ATV. Detailed methodology is provided in the Supplementary Materials [34,35,36,37,38]. PK parameters for Chinese HVs and ESRD patients are summarized in Table S7.

### 2.4. Validation of PBPK Drug Models in HVs

All drugs were fully validated using PK data collected from therapeutic doses (except for DABE and MDZ [29,39]), microdose [27,40,41], drug–drug interaction (DDI, co-administered with rifampicin, itraconazole, or clarithromycin) [40] and pharmacogenomic (PGx) studies (*SLCO1B1* and *ABCG2*) [34,35,36]. The age range, female proportion, and dosing regimen were set to match the designs of corresponding literature or clinical trials. The unbound fraction (*f*_u_) of substrates in ESRD patients were calculated based on the measured ratios between ESRD patients and HVs [41,42], except for DABE, for which plasma protein binding is low and not significantly altered in ESRD patients (Table S8) [41,42,43]. The perpetrator drug models were adopted from the Simcyp inhibitor library (Table S9). For PGx studies, phenotype definitions followed the Clinical Pharmacogenetics Implementation Consortium (CPIC) guidelines [37], and the details are listed in Supplementary Materials.

The predictive performance of PBPK model was evaluated using the ratio of predicted-to-observed AUC and C_max_ (Pre/Obs ratio). Similarly, for DDI studies, the AUC ratio with or without perpetrator (AUCR_w/w-o_) was calculated, and the ratio of predicted-to-observed AUCR_w/w-o_ was used to assess the predictive performance. The predictive performance was considered acceptable when the Pre/Obs ratio fell within a 0.5–2-fold range.

### 2.5. Development of ESRD Population Model

Global Sensitivity Analysis (GSA) of drug exposure for the five substrates was performed using the Morris method in Simcyp, which identified human serum albumin (HSA), hematocrit, α-acid glycoprotein (AGP), kidney size, kidney-size density, liver density, cardiac output scalar, and gastric and drug mean residence time as sensitive factors for AUC_0–t_ (Figure S2). Based on the Simcyp built-in “Sim-Chinese Healthy Volunteers” population model, we constructed a novel ESRD population model by integrating the following sensitive physiological and pathological parameters in ESRD patients: (1) demographic data, including gender, age, height, and weight obtained from the China Kidney Disease Network (CK-NET) 2016 [44]; (2) serum creatinine levels in ESRD patients sourced from CK-NET 2016 and estimated glomerular filtration rate (eGFR) calculated using the CKD-EPI equation [45]; (3) HSA, hematocrit, and AGP data collected from outpatient ESRD patients at Peking University Third Hospital between 2013 and 2023; (4) kidney size and density were adopted from the “Sim-Renal Impairment_Severe” model, the closest physiological stage to ESRD; (5) liver density and cardiac output were adopted from the “Sim-Chinese healthy volunteers” model due to physiological similarities between HVs and ESRD patients [46]; (6) gastric and drug mean residence time were adjusted from the built-in parameters of the “Sim-Chinese Healthy Volunteers” model by applying the ratio between the “Sim-Renal Impairment_Severe” and “Sim-Healthy Volunteers” models.

Then, a back-calculation method was employed to quantify the abundance of each DMET in ESRD patients, which was initially assumed to be consistent with that in HVs and modified according to observed changes in drug exposure in ESRD patients. The AUC ratio between ESRD patients and HVs (AUCR_ESRD/HV_) was calculated to assess the relative change in DMET function, with an acceptance criterion defined as the Pre/Obs ratio falling within 0.8- to 1.25-fold. MDZ (CYP3A4), DABE (intestine P-gp), and PTV (OATP1B1/3) were firstly used to evaluate the effects of ESRD on single elimination pathways. Subsequently, intestinal BCRP abundance was estimated using RSV by fixing OATP1B1/3 abundance according to PTV and adjusting renal OAT3 function to 24.6% of that in HVs based on previous studies [47]. Finally, ATV was utilized to validate the combined effects of functional changes in CYP3A4, OATP1B1/3, P-gp, and BCRP.

### 2.6. Application of ESRD Population Model in Evaluating ADR Risks

The constructed ESRD population model was utilized to simulate untested scenarios. Drugs commonly prescribed for ESRD patients were used as victims, including lipid-lowering drugs PTV, RSV, and ATV. In addition, the angiotensin II receptor blocker valsartan that is hepatically transported by OATP1B1 and OATP1B3 (parameters summarized in Table S10) was also included [48]. For inhibitors, single-dose rifampin (inhibitor of OATP1B1 and OATP1B3), itraconazole (strong inhibitor of CYP3A4 and P-gp), clarithromycin (strong inhibitor of CYP3A4, P-gp, OATP1B1, and OATP1B3), ritonavir (strong inhibitor of CYP3A4), and verapamil (moderate inhibitor of CYP3A4 and P-gp) were included.

Firstly, muscle exposure to PTV, RSV, and ATV was simulated under both single- and multiple-dose regimens to evaluate their potential myotoxicity. Then, simulations were performed for victim drugs co-administrated with perpetrator drugs (rifampin, itraconazole, clarithromycin, ritonavir, and verapamil) in both HVs and ESRD patients, respectively (Table S9). Drug combination scenarios are shown in Figure S3. Each simulation was conducted using a virtual population consisting of 10 trials. Each trial includes 10 Chinese HVs or ESRD patients (50% female) between 20 and 70 years old.

### 2.7. Application of ESRD Population Model in Exploring Individual Influencing Factors

For each patient in the microdose cocktail clinical trial, individual concentration–time curves of each drug were simulated by PPK base model and ESRD PBPK model with corresponding demographic, physiological, and genotypic parameters [27]. PPK and PBPK simulations were performed over a 72 h period. Apparent clearance of PPK (CL/F_PPK_) and PBPK simulated concentration–time curve (CL/F_PBPK_) were calculated by non-compartmental analysis (NCA).

Gut microbiome and physiological parameters that were determined in a previous study [27] and not included in the PBPK population model (alanine aminotransferase, aspartate aminotransferase, total bilirubin level, etc.) were subjected to correlation analysis with CL/F. Variables significantly correlated with the ratio of CL/F_PBPK_ and CL/F_PPK_ (*p* < 0.05) were selected for multiple linear regression (MLR) analysis using the least absolute shrinkage and selection operator (LASSO) algorithm. The most predictive variables were selected by the minimum λ (λ min).

### 2.8. Software

All PBPK-related studies were conducted using the Simcyp Simulator (Version 22; Certara, Sheffield, UK). NCA was performed using Phoenix WinNonlin (version 8.3.3). The linear up log down method was employed for calculations. PPK simulation was performed using non-linear mixed-effects modeling software (NONMEM, version 7.2). The correlation coefficient analysis and LASSO regression were performed with R (version 4.1.3).

## 3. Results

### 3.1. Validation of Drug Models in HVs

**Figure 2:**
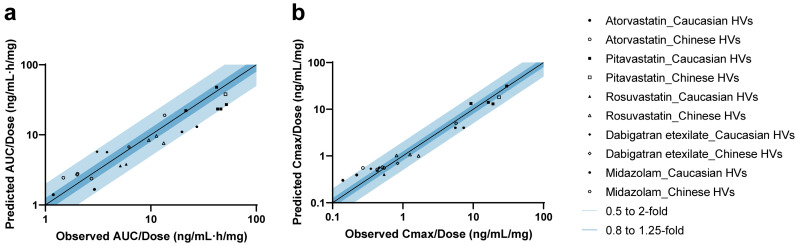
Predicted-to-observed ratios of PK parameters in healthy volunteers. Summary of predicted-to-observed ratios for AUC (**a**) and C_max_ (**b**) across five drugs. Mean or geometric mean value was used based on the data from each research paper. The black line represents the line of unity, while the dark and light blue shaded areas indicate ratio intervals of 0.8–1.25-fold and 0.5–2-fold, respectively.

A total of 11 studies were included for the validation of five drug models (Figure S4). In addition, studies from the clinical pharmacology review for PTV (LIVALO) from the FDA were also included [49]. For all drugs, the observed concentration–time data aligned well with the predicted PK profiles in both Caucasian and Chinese HVs under therapeutic dose (Figure S5) [49,50,51,52,53,54] and microdose (Figure S6) [40,41] administration. As shown in Figure 2 and Table S11 [27,40,41,49,50,51,52,53,54], 95.7% (22/23) of predicted AUC values and 91.3% (21/23) of predicted C_max_ values fell within the 2-fold range. The MDZ model slightly underestimated the AUC for one Caucasian microdose cohort (Pre/Obs ratio: 0.48), whose observed exposure was 39% higher compared to another cohort of the same dose and ethnicity. For ATV, the model predicted the AUC of most cohorts (4/6) across ethnicities well, although C_max_ values for two microdose cohorts were slightly overestimated (Pre/Obs ratios: 2.16 and 2.05, respectively).

Then, all drug models were validated using DDI data involving co-administration with rifampicin, itraconazole, and clarithromycin [40]. The dosing regimens and predictive performance are listed in Table S12 [40]. The DDI magnitude was slightly overestimated for PTV co-administered with clarithromycin (Pre/Obs ratio: 2.09). Systematic underestimation was observed for DABE when co-administered with P-gp inhibitors. This discrepancy may be attributed to the non-linearity of intestinal P-gp-mediated DDIs under microdose conditions, likely due to the saturability of P-gp efflux capacity, as previously discussed [41].

Given that *SLCO1B1* and *ABCG2* polymorphisms significantly affect the PK parameters of their respective substrates [31,34,55], we further validated the statin models using clinical PGx data. The corresponding Pre/Obs ratios for different transporter phenotypes are summarized in Tables S13 and S14, with values falling within the acceptable 2-fold range, except for three cohorts with limited sample sizes (N ≤ 3).

### 3.2. Development of ESRD Population Model

Physiological parameters associated with drug disposition were collected and incorporated into the development of the Chinese ESRD population model (Table 1 and Table S15 [44]). The mean serum creatinine concentration in ESRD patients was 721.69 ± 321.92 µmol/L, and the predicted serum creatinine concentrations closely matched the observed values in ESRD patients from our clinical trial (Figure S7). Furthermore, compared with HVs, hematocrit and HSA decreased by 19.26–26.93% and 27.13–28.99%, respectively, whereas AGP levels increased by 52.09–77.73%.

**Figure 3:**
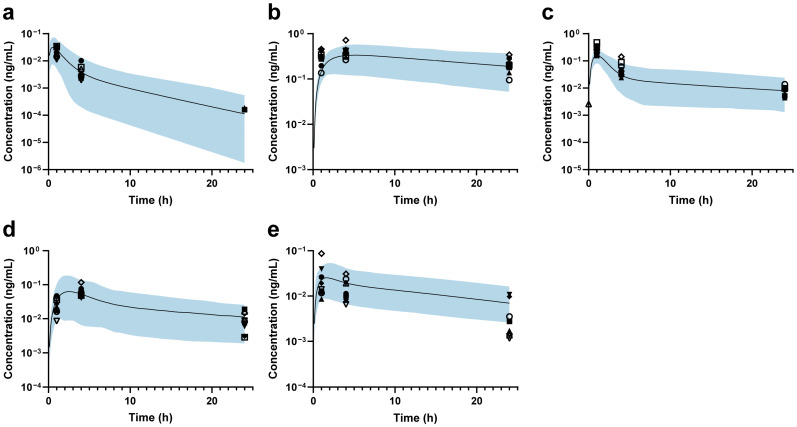
Plasma concentration–time profiles in ESRD patients. (**a**) Midazolam; (**b**) dabigatran; (**c**) pitavastatin; (**d**) rosuvastatin; (**e**) atorvastatin. The black line represents the predicted mean plasma concentration–time profile, while the shaded area represents the 90% prediction intervals. The black markers indicate individual observed data for each Chinese ESRD patient.

Then, the back-calculation method was applied to estimate changes in DMET functions in ESRD patients. For MDZ, the predicted and observed AUCR_ESRD/HV_ values showed good agreement, suggesting that hepatic CYP3A4 abundance remains unchanged (Figure 3a). The predicted DAB AUCR_ESRD/HV_ aligned well with the observed data when a 34% reduction in intestinal P-gp abundance was applied (Figure 3b). Based on PTV exposure, the abundance of OATP1B1 and OATP1B3 was estimated to decrease by 75% in ESRD patients (Figure 3c). After setting the abundance of OATP1B1/3 to 25% and renal OAT3 to 24.6% of that in HVs [47], a 100% increase in ileum BCRP abundance was applied to accurately predict RSV exposure in ESRD patients (Figure 3d). Finally, the combined effects of modified abundances of hepatic OATP1B1/3, CYP3A4, intestinal P-gp, and BCRP were further validated using ATV, demonstrating good agreement between the predicted and observed AUCR_ESRD/HV_ values (Figure 3e). Accordingly, the final ESRD population model was constructed (the optimization process is shown in Figure S8), and the modified DMET parameters are listed in Table 2.

### 3.3. Application of ESRD Population Model in Evaluating ADR Risks

**Figure 4:**
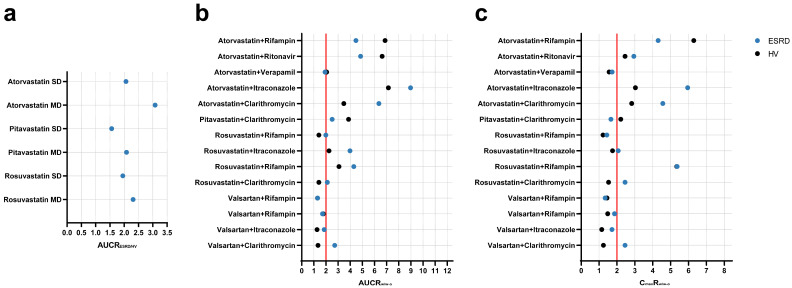
Simulated geometric mean ratios of muscle exposure and drug–drug interactions between healthy volunteers and ESRD patients. (**a**) muscle AUC; (**b**) AUC ratio; and (**c**) Cmax ratio. AUCR_ESRD/HV_ area under the curve ratio between ESRD patients and HVs; AUCR_w/w-o_ area under the curve ratio with/without perpetrator; C_max_R_w/w-o_ maximum plasma concentration ratio with/without perpetrator; SD single-dose; MD multiple-dose; HV, healthy volunteers; ESRD, end-stage renal disease patients.

The PBPK population models for HVs and ESRD patients were used to simulate different scenarios to assess potential ADR risks. Firstly, simulations were conducted to explore the muscle exposure of three statins (PTV, RSV, and ATV) in HVs and ESRD patients under single-dose and multiple-dose regimens. As shown in Figure 4a and Table S16, significant increases were observed in muscle AUC for all three statins in ESRD patients, especially under multiple-dose regimens (AUC increased by more than 2-fold). Moreover, simulations were performed to evaluate the additional risk introduced by ESRD by comparing DDI magnitudes in ESRD patients and HVs. As shown in Figure 4b,c and Table S17, ESRD patients exhibited greater DDI magnitudes. For instance, the DDI magnitude of valsartan-clarithromycin in ESRD patients were 2-fold of that observed in HVs. Taken together, these simulations indicate potential ADR risks in ESRD patients that were not evident in HVs.

### 3.4. Application of ESRD Population Model in Exploring Individual Influencing Factors

**Figure 5:**
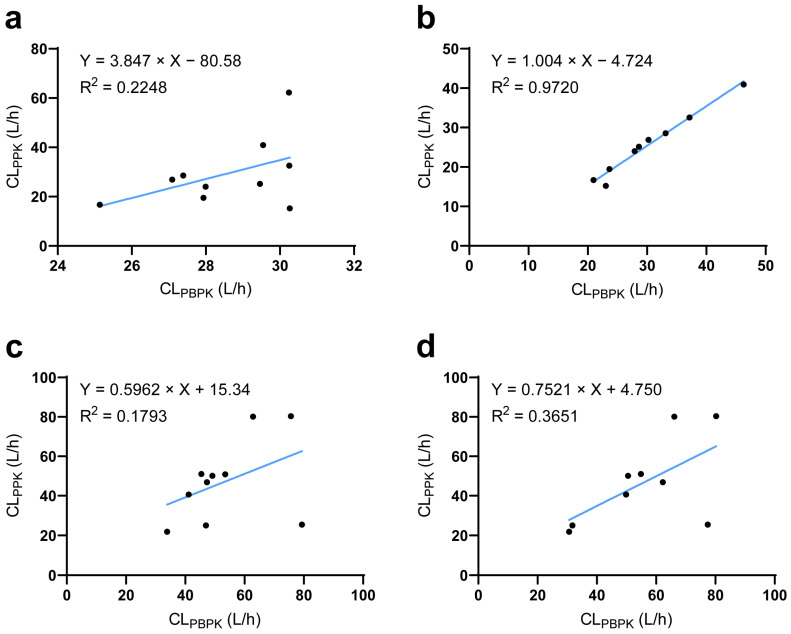
Comparison of predicted and observed clearance at the individual level. (**a**) The relationship between PBPK- and PPK-predicted DAB clearance before or (**b**) after corrected by LASSO analysis. (**c**) The relationship between PBPK- and PPK-predicted RSV clearance before or (**d**) after corrected by LASSO analysis. CL_PPK_ population pharmacokinetic model-predicted clearance; CL_PBPK_ physiologically based pharmacokinetic model-predicted clearance.

The established ESRD PBPK model integrated demographic parameters, phycological parameters, and genotypes and could thereby be used to simulate individual PK profiles. PPK is a well-accepted technique to reflect individual PK characteristics. Thus, using a PPK-simulated individual PK profile as reference [27], we found that the ESRD PBPK model could not capture individual PK characteristics of DAB and RSV well (Figure 5a,c). To solve this problem, we investigated potential factors influencing individual clearance by LASSO regression. The ratio between CL/F_PBPK_ and CL/F_PPK_ was regarded as the differences between predicted and “observed” data that could not be explained by PBPK integrated parameters. LASSO analysis identified the abundance of *Clostridium XVIII* and *Escherichia* genus as significant influencing factors for DAB, the substrate of intestine P-gp (Equation (1)). For RSV, aspartate aminotransferase and total bilirubin were identified as significant factors, consistent with the liver being its major elimination route. In addition, *Bacteroides* and *Megamonas* genus abundances were also identified to significantly impact RSV CL/F. For MDZ, PTV, and ATV, no significant influencing factors were identified. The correlation between PBPK- and PPK-predicted drug clearance was significantly improved after integrating the novel influencing factors (Figure 5b,d).

(1) $$\frac{CL}{F\left(DAB\right)}=1.11+0.152\times Clostridium_{XVIII}+0.0558\times Escherichia$$

(2) $$CL/F\left(RSV\right)=1.67-0.0581\times AST+0.0347\times TBIL-0.00596\times Bacteroides+1.847\times Megamonas$$

*Clostridium_XVIII*, *Escherichia*, *Bacteroides*, and *Megamonas* represent their relative abundance of their respective bacterial genera in the gut microbiome, while AST and TBIL refer to serum aspartate aminotransferase and total bilirubin level, respectively.

## 4. Discussion

Accumulating evidence demonstrated the significant impact of CKD on drug exposure [5,6,7] with large IIV [11] through multiple mechanisms. Characterizing the synergistic effects of CKD, especially at individual level, remains to be a significant challenge. The PBPK model is advantageous in integrating the alterations of demographic, physiological, and genotypic parameters, as well as DMET functions. However, when multiple DMET functions require simultaneous adjustment, high-quality clinical data with consistent demographic, physiological, and genotypic backgrounds are needed to minimize the influence of IIVs. In this study, we used PK data of five substrate drugs collected from a single Chinese ESRD patient cohort to establish a novel PBPK population model.

Systematic analysis of 18 CYP3A4 sensitive substrate drugs (AUC ratio attributable to hepatic CYP3A4 inhibition greater than 3-fold) revealed no apparent relationship between the severity of CKD and CYP3A4-mediated clearance [7]. Midazolam is recommended by the FDA as a probe for CYP3A4 function, as its exposure increased 10- to 15-fold when co-administrated with ketoconazole or itraconazole (CYP3A4 strong inhibitors) [56]. Consistent with our study, MDZ exposure is decreased in ESRD patients compared to healthy subjects [27,41] due to increased unbound clearance attributable to changes in *f*_u_ [42]. In our study, intestinal P-gp function was estimated to decrease by 34%. This magnitude is within the 30% reduction in intestine P-gp efflux of rhodamine 123 in chronic renal failure rats [57] and the 40% reduction in P-gp efflux activity in severe CKD patients [41]. A previous study has indicated that OATP1B function decreases as kidney function declines [6] and reaches a 60% reduction in patients with severe CKD [30]. This aligns with the 75% reduction of OATP1B abundance in ESRD patients in our study. We could not assess the respective impact of ESRD on OATP1B1 and OATP1B3 individually since all the statins used in this study are dual substrates of both transporters [58]. Instead, we assumed that the functions of OATP1B1 and OATP1B3 change synchronously with the same magnitude, as has been done in previous studies [30,59,60]. In addition to being an OATP1B substrate, RSV is also recommended as a probe for evaluating BCRP function, as its exposure is significantly associated with *ABCG2* polymorphism [61]. Studies in CKD rats have shown that BCRP expression is up-regulated in the ileum (~75%) while remaining unchanged in the liver and other intestinal regions [62,63]. Consistently, after fixing OATP1B abundance, our study suggested that ileum BCRP abundance is up-regulated in a comparable magnitude (100%), whereas changes in other organs are minimal. Finally, after all DMET functions were modified, the PBPK model could predict the PK profile of ATV well, which is the substrate of CYP3A4, OATP1B, BCRP, and P-gp, further validating the predictive performance of our model.

Our simulations provide critical insights into the magnitude of DDIs in patients with ESRD, revealing that these interactions are often more pronounced than in HVs due to ESRD-induced changes in DMETs. These alterations can lead to greater exposure increases for substrates such as statins when co-administered with inhibitors. For example, our simulations demonstrated that muscle exposure of all three statins increased by more than 2-fold in ESRD patients compared to HVs after multiple administrations, especially for ATV (3.1-fold). Moreover, a 2-fold greater DDI magnitude was observed for valsartan-clarithromycin in ESRD compared to HVs. The additionally increased exposure is particularly concerning given that statins are among the most commonly prescribed medications in CKD patients [64], where statin-associated muscle symptoms (SAMS) represent the major ADR, with an incidence ranging from 10% to 29% [65]. The occurrence of SAMS has been suggested to be concentration-dependent due to direct statin myotoxicity [37]. Our findings are consistent with a recent pharmacovigilance study that identifies ATV as the statin with the highest risk for rhabdomyolysis, with clarithromycin frequently reported as a co-reported drug in such cases [66]. Therefore, the simulations of drug combinations revealed potential DDI risks that were not observed in HVs, underscoring the need for the assessments to guide personalized dosing and mitigate toxicity in ESRD patients.

Although the PBPK model is advantageous in predicting the PK profiles at a population level, it may have limitations when applied to individual-level predictions. In this study, we employed a machine learning (ML) method to identify the significant factors influencing individual drug clearance and to quantify their contributions. Unlike our previous study, which used PPK model-predicted individual CL/F as dependent variable, the ratio between CL/F_PBPK_ and CL_PPK_ was applied in this study [27]. Given that CL/F_PBPK_ is calculated by the simulation results that have already integrated multiple demographic, physiological, and genotypic parameters, this approach enabled us to identify additional variables that further explain IIVs, such as gut microbiome. A recent clinical study revealed that specific bacterial taxa and amplicon sequence variant could explain the IIV in tacrolimus exposure in in kidney transplant patients [67]. Further study demonstrated that the altered absorption of tacrolimus is associated with the interaction between gut microbiome and intestine P-gp expression [68]. In our study, the abundance of Clostridium_XVIII and *Escherichia* genus were identified as potential influencing factors to explain absorption IIV mediated by intestinal P-gp. Notably, *Bacteroides* genus abundance, a newly identified influencing factor for RSV, was reported to explain the heterogeneity in both statin responses and adverse effects [69], possibly by affecting statin metabolism [70]. Further investigations are warranted to validate the role of these influencing factors in individual drug disposition. Nevertheless, our study provides a practical strategy to establish individualized PBPK models.

There are still several limitations in our study. First, the data we used to establish population model are from a clinical trial using microdose administration. The extrapolation between microdose and therapeutic dose needs further investigation. For instance, drug model validation indicated that intestinal P-gp saturation may lead to the non-linearity of DAB exposure when co-administrated with perpetrators. Furthermore, endogenous biomarkers, such as coproporphyrin-I and pyridoxic acid, are well acknowledged as a valuable tool for assessing transporter function in CKD patients [71,72]. Incorporating endogenous biomarker levels in ESRD patients could not only be used to validate the functional changes of OATP1B from a complementary perspective but also to inform new transporter function that was not explored in our current study (e.g., OAT1/3). At last, DDDI should be evaluated for the drug combinations commonly prescribed in clinical practice to better indicate ADR risks in real-world settings.

## Figures and Tables

**Table 1:** Physiological parameters for the Chinese ESRD population model.

Physiological Parameter	Gender	Value
Demographic		
Age-BH ^a^	M	BH = 176.18 – 0.2623 × age + 0.0016 × age^2^
	F	BH = 161.15 – 0.1405 × age + 0.0005 × age^2^
BW-BH ^a^	M	BW = exp(1.9619 + 0.01314 × BH)
	F	BW = exp(2.442 + 0.01000 × BH)
Kidney		
Serum creatinine mean (CV%) ^a^	M	Less than 50: 800 (38.4) Greater than 50: 674.74 (42.45)
	F	Less than 30: 800 (27.85) Greater than 30: 666.80 (33.44) Greater than 60: 533.32 (34.01)
Kidney-size parameters ^b^		Volume Baseline: 5.7 BW coefficient: 1.04 BH coefficient: 29.8 CV%: 23.4 Density (g/L): 1050
GI tract		
Gastric residence time (h) (CV%) ^c^		Stomach (fasted): 0.80 Stomach (fed): 3.35 Colon: 30
Fluid and dissolved drug mean residence times for whole colon (h) (CV%) ^c^	M	36.72
	F	50.14
Blood		
Hematocrit (%) (CV%) ^d^	M	33.1 (17.18)
	F	32.7 (16.49)
AGP (g/L) (CV%) ^d^	M	1.0388 (39.00)
	F	1.0948 (34.08)
HSA (g/L) (CV%) ^d^	M	35.12 (27.38)
	F	34.43 (26.75)

^a^ The equations and serum creatinine concentrations were obtained from the China Kidney Disease Network (CK-NET) 2016 annual data report [44]. ^b^ Kidney-size parameters were adopted from the “Sim-Renal Impairment_Severe” model in Simcyp (Version 22). ^c^ Based on the built-in parameters of the “Sim-Chinese Healthy Volunteers” model, gastric residence time and mean residence times were calculated according to the increased ratio between the “Sim-Renal Impairment_Severe” and “Sim-Healthy Volunteers” models. ^d^ Data were collected from outpatient ESRD patients at Peking University Third Hospital (between 2013 and 2023). BH body height (cm); BW body weight (kg); GI Tract gastrointestinal tract; AGP α-acid glycoprotein; HSA human serum albumin; M male; F female; CV coefficient of variation.

**Table 2:** Functional changes in the Chinese ESRD population model. The default values are from Simcyp (Version 22), and the values in blue background represent the modified values.

	Tissue	Chinese Healthy Volunteers					Chinese ESRD Patients				
		AA or RA ^c^	EM/T	PM/T	IM/T	UM/T	AA or RA	EM/T	PM/T	IM/T	UM/T
CYP3A4	Liver	120	1	0	0	0	120	1	0	0	0
	Intestine	57.3	1	0	0	0	57.3	1	0	0	0
	Colon	2.58	1	0	0	0	2.58	1	0	0	0
OATP1B1 ^a^	Liver	3.1	0.584	0.21	0.4	0.81	3.1	0.146	0.0525	0.1	0.2025
OATP1B3 ^a^	Liver	3.08	1	0	0	0	3.08	0.25	0	0	0
P-gp	Liver	0.246	1	0	0	0	0.246	1	0	0	0
	Jejunum	0.4	1	0	0	0	0.4	0.66	0	0	0
BCRP	Ileum	0.78	1	0.37	0.67	0	1.56	1	0.37	0.67	0
	Liver	0.103	1	0.37	0.67	0	0.103	1	0.37	0.67	0
	Renal	0.120	1	0.37	0.67	0	0.120	1	0.37	0.67	0
OAT3 ^b^	Renal	1.320	1	0	0	0	1.320	0.246	0	0	0

^a^ The functions of OATP1B1 and OATP1B3 were assumed to change synchronously with identical folds. The impact of polymorphisms on *SLCO1B1* activity in ESRD patients was assumed to result in the same proportional reduction from their corresponding baseline values. ^b^ Based on the CK-NET 2016 report, which indicated a mean eGFR of 10.81 mL/min/1.73 m^2^ in ESRD patients [44], an OAT-mediated secretory function was calculated using the following equation [47]: $OAT\left(\%\right)=\left(1-e^{\left(-{\left(\frac{eGFR}{43.9}\right)}^{0.9}\right)}\right)\times 100\%$. ^c^ AA applies to all listed enzyme and transporters except for BCRP in ileum. AA absolute abundance in metabolizing enzyme (pmol/mg-protein) or transporters (pmol/10^6^ hepatocytes); RA relative abundance of BCRP in ileum compared with jejunum I; EM/T extensive metabolizer or transporter; PM/T poor metabolizer or transporter with reduced activity; IM/T intermediate metabolizer or transporter with intermediate reduced activity; UM/T ultra-rapid metabolizer or transporter with increased activity.

## Data Availability

Data is available on request.

## Supplementary Materials

The following supporting information can be downloaded at: https://www.mdpi.com/article/10.3390/pharmaceutics17081078/s1. Figure S1. Hepatic uptake scaling factors calibrated to fit *SLCO1B1* pharmacogenomic data for pitavastatin. Figure S2. Influence of input physiological parameters on AUC_0-t_ for substrate drug models. Figure S3. Drug combination scenarios of prospective simulation in ESRD patients. Figure S4. Literature search strategy for PBPK drug model validation in healthy volunteers. Figure S5. PBPK model-predicted and literature-observed concentrations in healthy volunteers (HVs) at therapeutic doses. Figure S6. PBPK model-predicted and observed concentrations in healthy volunteers (HVs) at microdose. Figure S7. Predicted and observed serum creatinine concentrations in Chinese ESRD patients. Figure S8. Comparison of probe drug exposure between PBPK predictions and observations. Table S1. Summary of drug-dependent parameters for the midazolam PBPK model. Table S2 [29]. Summary of drug-dependent parameters for the dabigatran etexilate PBPK model. Table S3 [29]. Summary of drug-dependent parameters for the dabigatran and dabigatran glucuronide PBPK models. Table S4 [30,31]. Summary of drug-dependent parameters for the pitavastatin PBPK model. Table S5 [26]. Summary of drug-dependent parameters for the atorvastatin PBPK model. Table S6 [33]. Summary of drug-dependent parameters for the rosuvastatin PBPK model. Table S7. Pharmacokinetics of five substrates in Chinese healthy volunteers and ESRD patients. Table S8. Changes in unbound fraction in ESRD patients. Table S9. Input K_i_ values for inhibitors and their metabolites from the Simcyp inhibitor library. Table S10. Summary of drug-dependent parameters for the valsartan PBPK model. Table S11. Summary of dose-normalized predicted PK parameters in healthy volunteers. Table S12. Simulated drug–drug interactions in Caucasian healthy volunteers. Table S13 [31,34,35]. The predicted PK parameters for *SLCO1B1* polymorphism phenotype in healthy volunteers. Table S14 [35,36]. The predicted PK parameters for *ABCG2* polymorphism phenotypes in healthy volunteers. Table S15. Physiological parameters of HVs and ESRD patients. Table S16. Simulation of muscle exposure in healthy volunteers and ESRD patients. Table S17. Simulation of drug–drug interactions in healthy volunteers and ESRD patients.
