# Supplementary material for: Development of PBPK Population Model for End-Stage Renal Disease Patients to Inform OATP1B-, BCRP-, P-gp-, and CYP3A4-Mediated Drug Disposition with Individual Influencing Factors
Source: Pharmaceutics. 2025 Aug 20;17(8):1078. doi: 10.3390/pharmaceutics17081078 (PMC12389332; doi:10.3390/pharmaceutics17081078)
Supplement: Supplementary file 1 [file pharmaceutics-17-01078-s001.zip › pharmaceutics-3743787-supplementary.pdf]

Article: Development of PBPK population model for end-stage renal disease patients to inform OATP1B-, BCRP-, P-gp-, and CYP3A4-mediated drug disposition with individual influencing factors

Journal: Clinical Pharmacokinetics

Authors: Yujie Wu[1,2#], Weijie Kong[1,2#], Jiayu Li[1,2], Xiaoqiang Xiang[3], Hao Liang[1,2*], Dongyang Liu[2,4*]

[1]Department of Nephrology, Peking University Third Hospital, Beijing, China.

[2]Drug Clinical Trial Center, Peking University Third Hospital, Beijing, China.

[3]Department of Clinical Pharmacy and Pharmacy Administration, School of Pharmacy, Fudan University, Shanghai, China.

[4]Institute of Medical Innovation, Peking University Third Hospital, Beijing, China

[#]These authors contributed equally to this work: Yujie Wu and Weijie Kong

Corresponding author:

Dongyang Liu and

E-mail: liudongyang@vip.sina.com

Hao Liang

E-mail: lianghao86@126.com

Supplementary Materials

Section I. Supplementary Methods

Clinical study design, drug concentration determination, and pharmacokinetic analysis

The healthy volunteer (HV) group ranged in age from 21 to 59 years, with 64.3% female participants, while the ESRD patient group ranged from 36 to 53 years, with 30% female participants. Blood samples were collected at 0 h (before dosing) and 0.5 h, 1 h, 2 h, 4 h, 8 h, 12 h, and 24 h after dosing for HVs and 0 h, 1 h, 4 h, and 24 h (before dialysis) for ESRD patients. Plasma concentrations of the five drugs were determined using a validated liquid chromatography coupled with mass spectrometer method [38]. The lower limits of quantification (LLOQ) were 0.5 pg/mL for MDZ, 10 pg/mL for DAB, and 1 pg/mL for statins. Pharmacokinetic parameters for each substrate were calculated using Pheonix WinNolin (Version 8.3.3). The area under the curve up to 24 hours ($AUC_{0\text{-}t}$) and C_{max} values were derived from non-compartmental analysis (NCA), applying the linear trapezoidal method for ascending concentrations and the logarithmic trapezoidal method for descending concentrations (linear up log down).

Validation of PBPK drug models in healthy volunteers

The validation of statin models was performed using pharmacogenomic (PGx) studies involving *SLCO1B1* (OATP1B1) and *ABCG2* (BCRP) polymorphisms [34–36]. Polymorphic transporter phenotypes of poor transporters (PTs), intermediate transporters (ITs), extensive transporters (ETs), and ultra-rapid transporters (UTs) were defined in Simcyp Simulator. Briefly, HVs with *SLCO1B1* diplotypes *1/*1 and *1/*14 were classified as ETs. Those with *1/*5 and *1/*15 were classified as ITs (transporter activity assumed to be 68% of that in ETs). Subjects with diplotypes *5/*5, *5/*15, and *15/*15 were classified as PTs (37% of ETs), while *14/*14 was classified as UTs (139% of ETs), in accordance with Clinical Pharmacogenetics Implementation Consortium (CPIC) guidelines [37]. Similarly, for *ABCG2* polymorphisms, HVs with the c.421 CC genotype were classified as ETs, those with CA genotype as ITs (67% of ETs), and individuals with AA genotype as PTs (37% of ETs).

Section II. Supplementary Figures

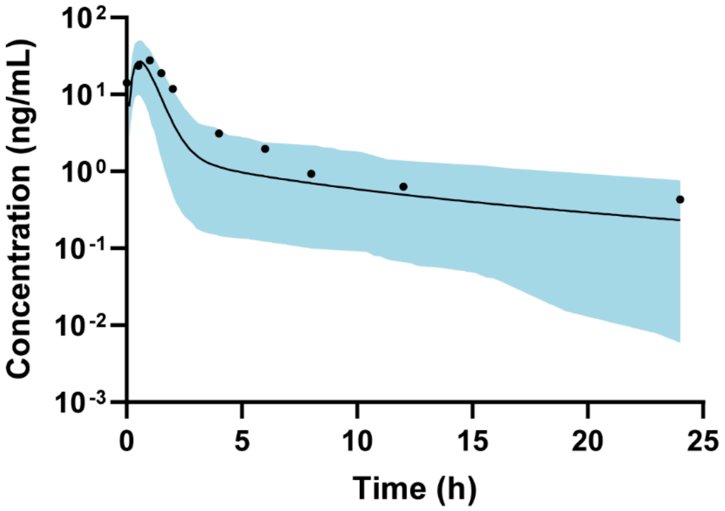

Figure S1. Hepatic uptake scaling factors calibrated to fit *SLCO1B1* pharmacogenomic data for pitavastatin.

The extensive transport phenotype was calibrated to fit *SLCO1B1* *1/*1 and *ABCG2* 421C/C observed data for Caucasian healthy volunteers [31]. The black line represents the predicted mean plasma concentration–time profile, the shaded area represents the 90% prediction intervals, and the black markers indicate observed data from the pharmacogenomic study.

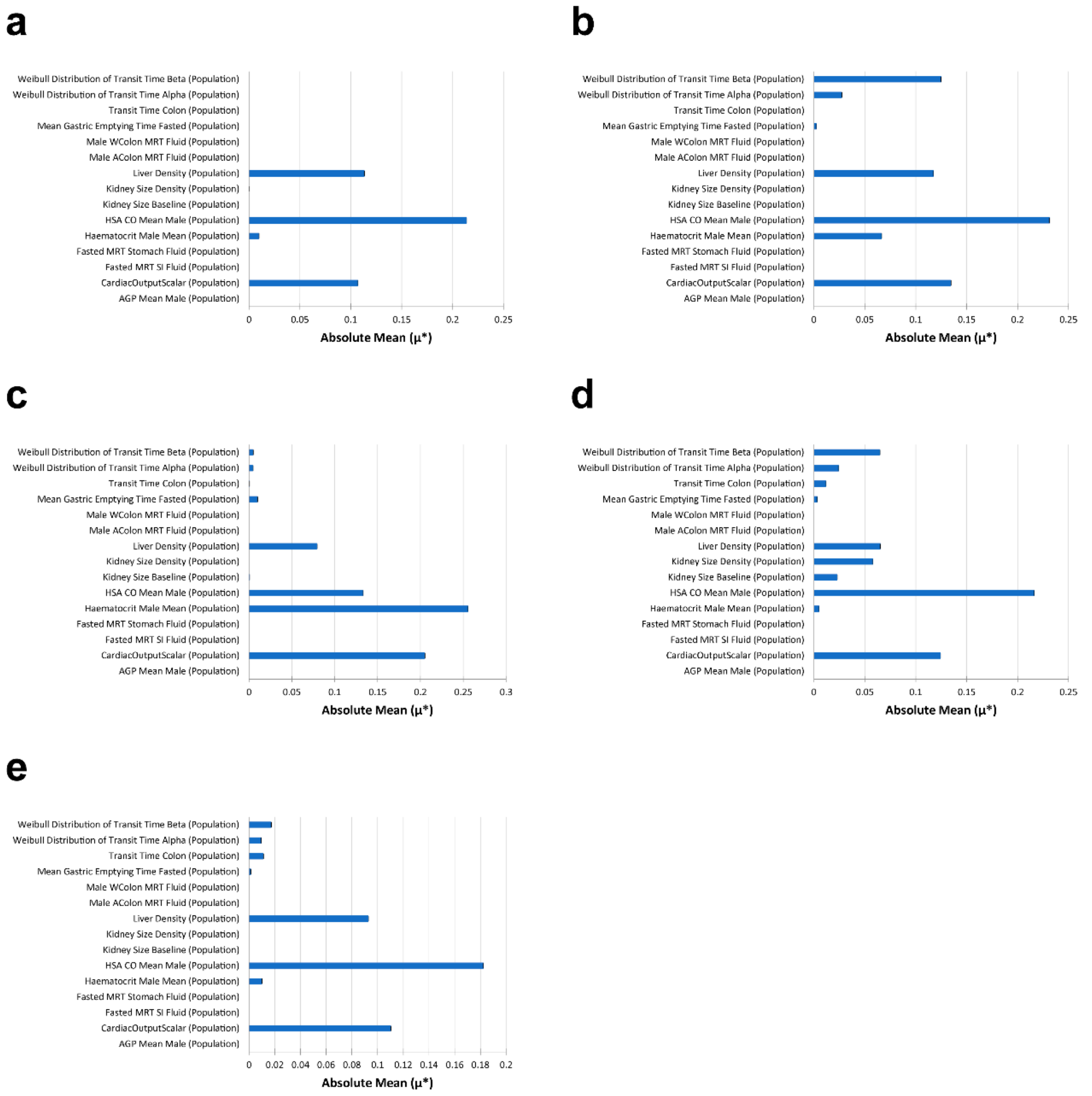

Figure S2. Influence of input physiological parameters on $AUC_{0\text{-}t}$ for substrate drug models. (a) Midazolam; (b) dabigatran etexilate; (c) pitavastatin; (d) rosuvastatin; (e) atorvastatin.

AGP α- acid glycoprotein; HSA human serum albumin; MRT mean residence times. The influence of input physiological parameters was assessed by the Global Sensitivity Analysis tool in Simcyp using the Morris method. A higher absolute mean (μ*) indicates a greater importance of the parameter on the model output.

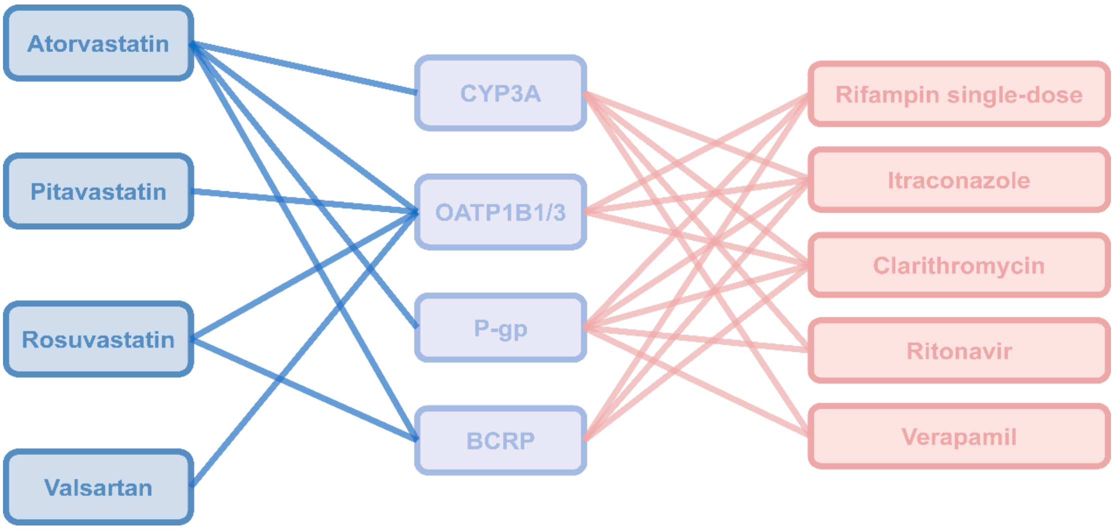

Figure S3. Drug combination scenarios of prospective simulation in ESRD patients.

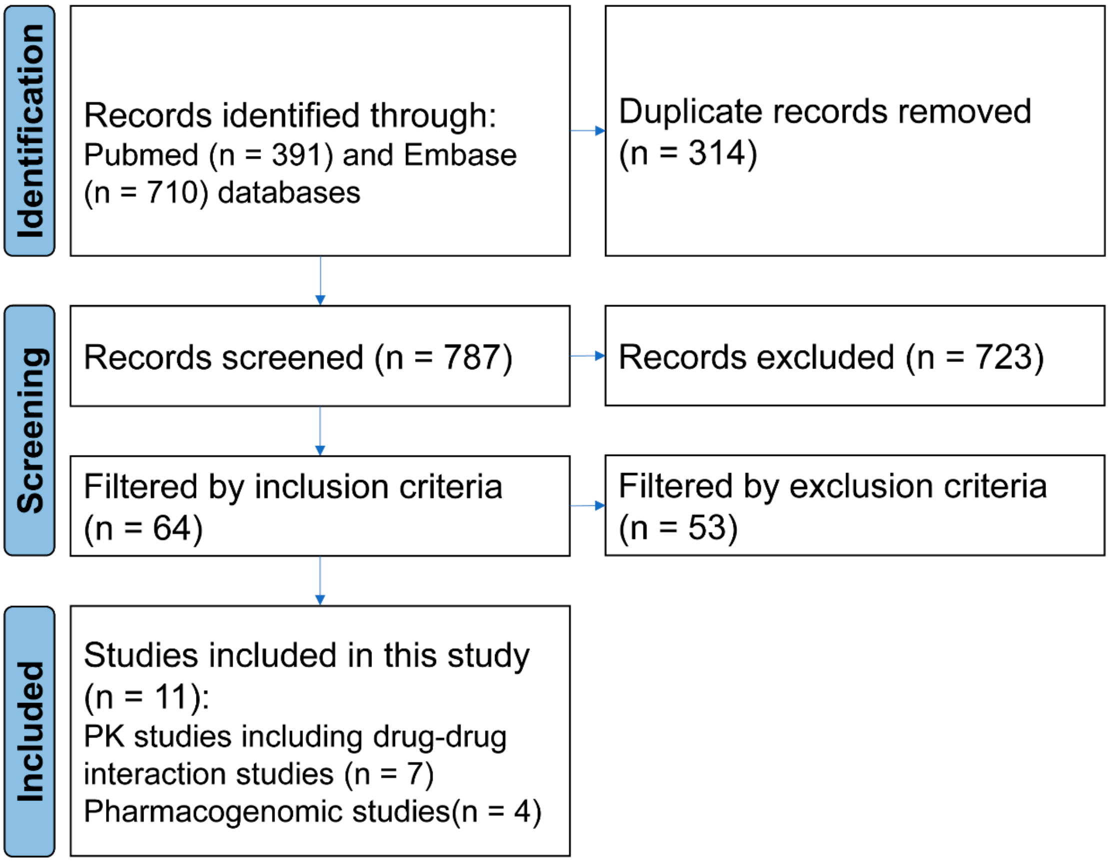

Figure S4. Literature search strategy for PBPK drug model validation in healthy volunteers.

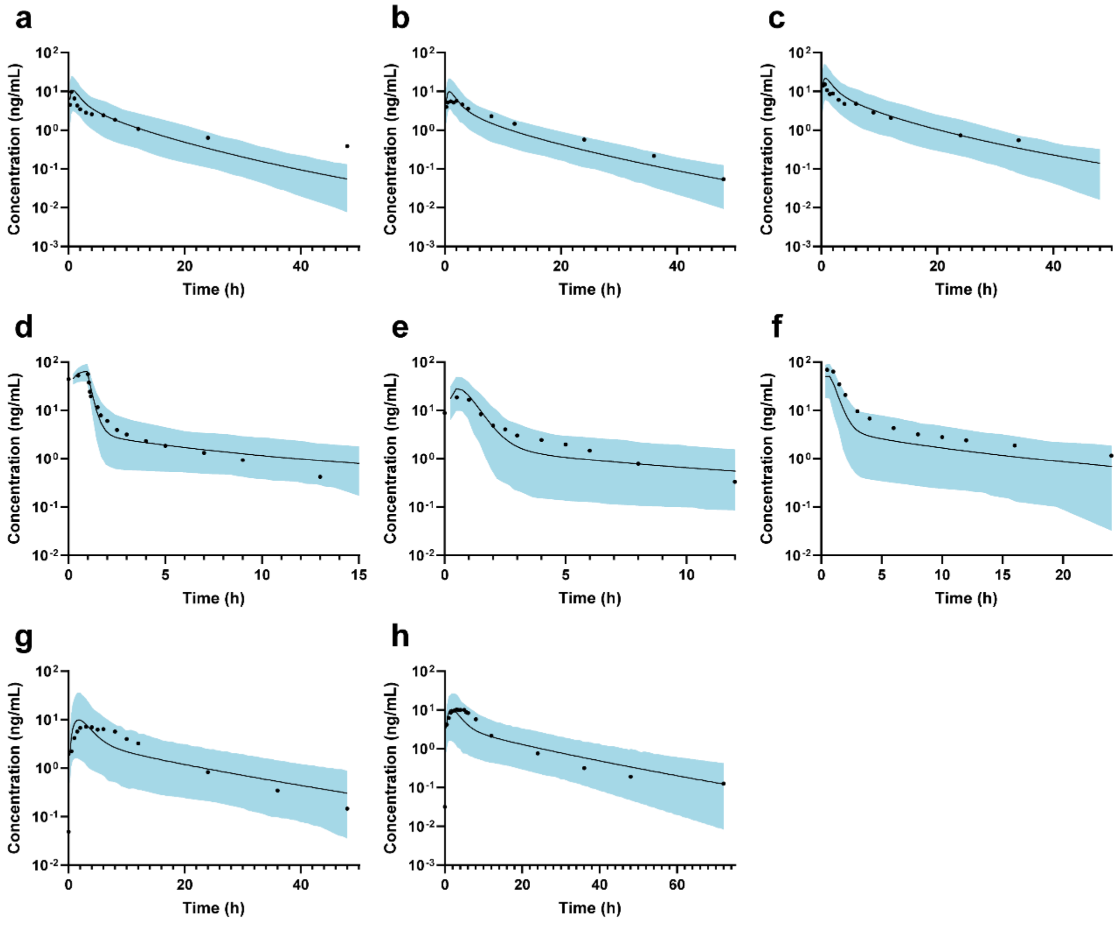

Figure S5. PBPK model-predicted and literature-observed concentrations in healthy volunteers (HVs) at therapeutic doses. Plasma concentration–time profiles for (a-c) atorvastatin in Chinese HVs[50–52]; (d-f) pitavastatin in Caucasian HVs, data from FDA Review for pitavastatin (LIVALO)[49]; and (g-h) rosuvastatin in Chinese HVs [53,54].

The black line represents predicted mean plasma concentration–time profile, the shaded area represents the 90% prediction intervals, and the black markers represent observed data from the literature.

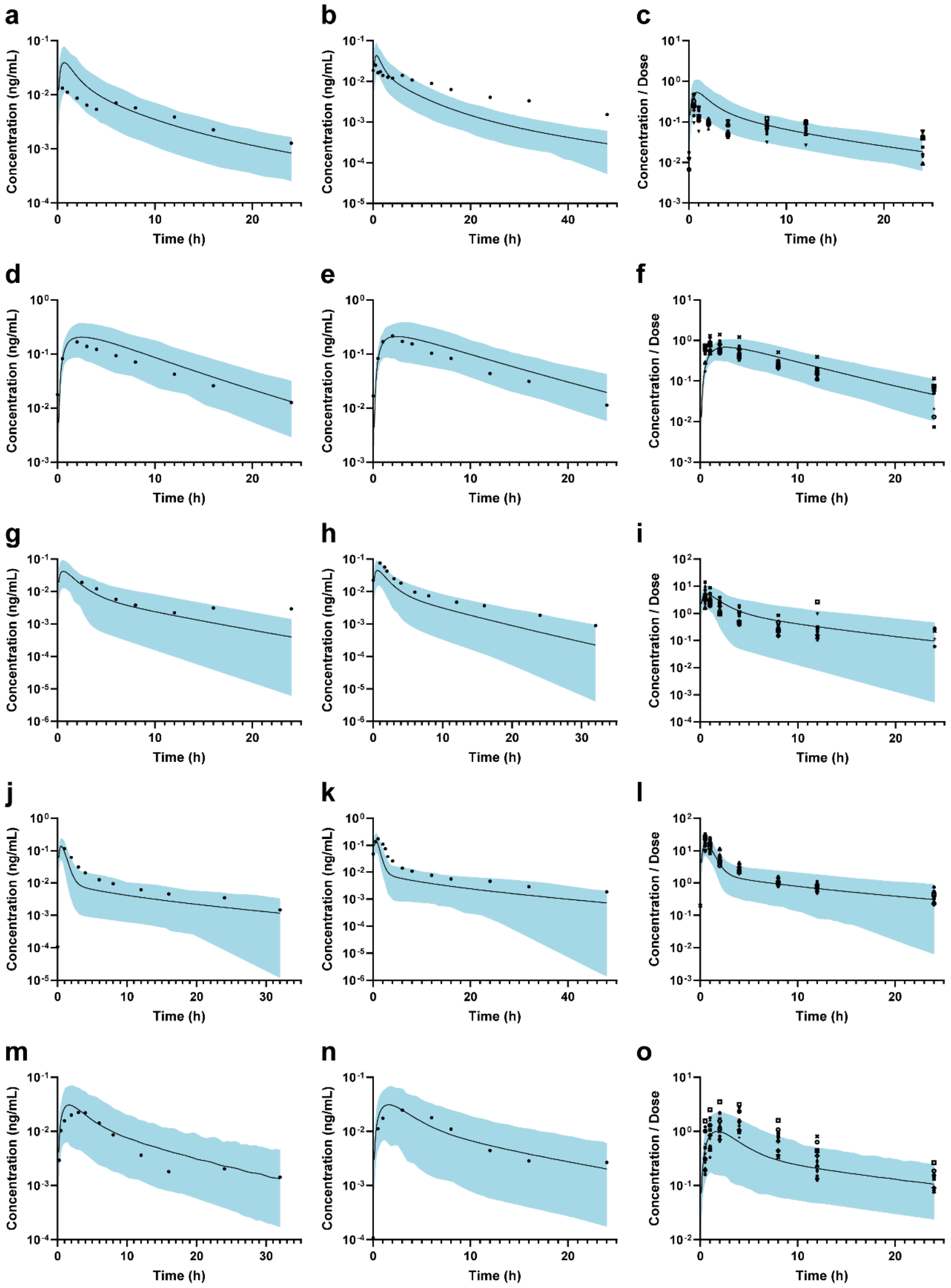

Figure S6. PBPK model predicted and observed concentrations in healthy volunteers (HVs) at microdose. Plasma concentration–time profiles for (a-b) atorvastatin in Caucasian HVs; (c) atorvastatin in Chinese HVs; (d-e) dabigatran in Caucasian HVs; (f) dabigatran in Chinese HVs; (g-h) midazolam in Caucasian HVs; (i) midazolam in Chinese HVs; (j-k) pitavastatin in Caucasian HVs; (l) pitavastatin in Chinese HVs; (m-n) rosuvastatin in Caucasian HVs; (o) rosuvastatin in Chinese HVs. The observed concentrations in Caucasian HVs were obtained from the literature [40,41].

The black line represents predicted mean plasma concentration–time profile, the shaded area represents the 90% prediction intervals, and the black markers represent observed data from the literature or clinical trials.

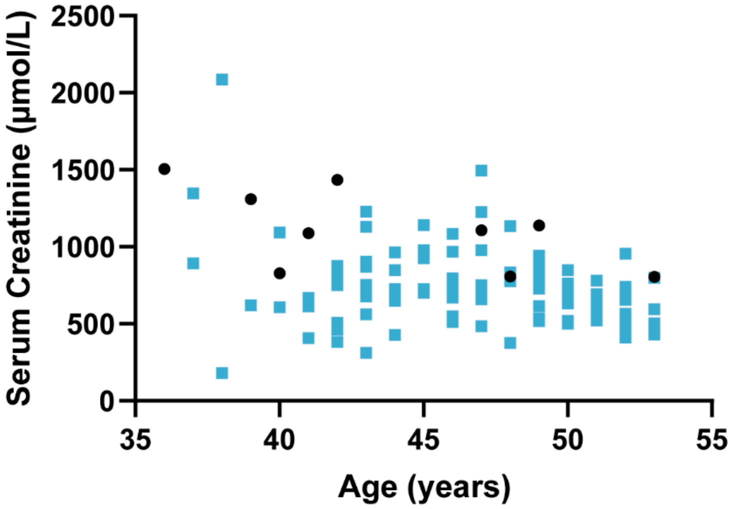

Figure S7. Predicted and observed serum creatinine concentrations in Chinese ESRD patients.
The blue points represent the predicted serum creatinine concentrations, while the black points represent the observed values from Chinese ESRD patients in the clinical trial.

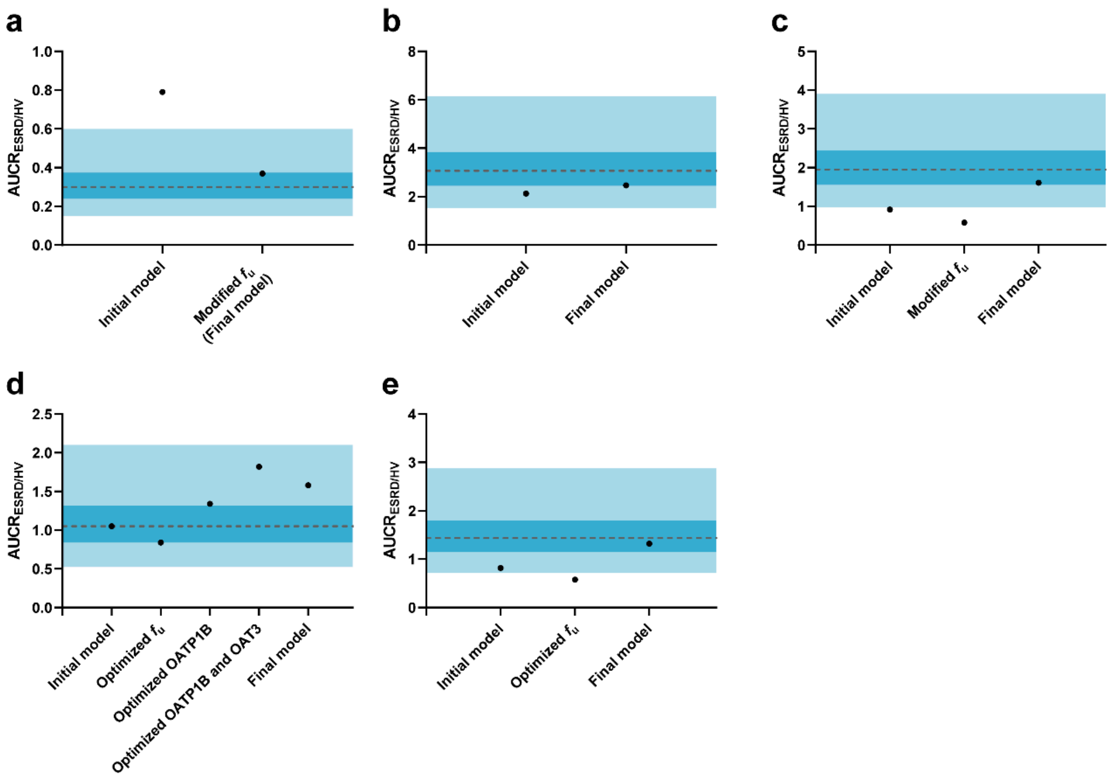

Figure S8. Comparison of probe drug exposure between PBPK predictions and observations. (a) Midazolam, the final model represents the ESRD population model with modified f_u, while hepatic CYP3A4 abundance remains unchanged; (b) dabigatran, the final model represents the ESRD population model with modified intestinal P-gp abundance; (c) pitavastatin, the final model represents the ESRD population model with modified f_u and hepatic OATP1B1/3 abundances; (d) rosuvastatin, the final model represents the ESRD population model with modified f_u, hepatic OATP1B1/3, and ileum BCRP abundances; (e) atorvastatin, the final model represents the ESRD population model with modified f_u, intestinal P-gp, BCRP, and hepatic OATP1B1/3 abundances, while hepatic CYP3A4 function remains unchanged.

$AUCR_{ESRD/HV}$ area under the curve ratio between ESRD patients and healthy volunteers; f_u unbound fraction.

The gray dashed line represents the observed AUCR$_{ESRD/HV}$ in Chinese ESRD patients, while the black solid dots represent the predicted AUCR$_{ESRD/HV}$. The dark blue shaded area indicates the ratio interval between 0.8- and 1.25-fold, and the light blue shaded area indicates the interval between 0.5- and 2-fold.

Section III. Supplementary Tables

Table S1. Summary of drug-dependent parameters for the midazolam PBPK model.

Parameters	Midazolam
**Physicochemical**	
Molecular weight (g/mol)	325.8
Log P	3.53
Compound type	Monoprotic base
pKa	6.00
Blood/plasma ratio	0.603
Fraction unbound	0.032
**Absorption**	
Absorption model	First order
$P_{eff,man}$ (10^{-4} cm/s)	6.0455
**Distribution**	
Distribution model	Minimal PBPK
Vss (L/kg)	0.88
**Elimination**	
CYP-mediated pathways (recombinant)	
CYP3A4 V_{max} (pmol/min/pmol) (1-OH)	5.23
CYP3A4 K_m (μM) (1-OH)	2.16
CYP3A5 V_{max} (pmol/min/pmol) (1-OH)	19.7
CYP3A5 K_m (μM) (1-OH)	4.16
CYP3A4 V_{max} (pmol/min/pmol) (4-OH)	5.2
CYP3A4 K_m (μM) (4-OH)	31.8
CYP3A5 V_{max} (pmol/min/pmol) (4-OH)	4.03
CYP3A5 K_m (μM) (4-OH)	38.4
UGT-mediated pathways (HLM)	
UGT1A4 V_{max} (pmol/min/mg)	445
UGT1A4 K_m (μM)	40.3
Renal clearance (L/hour)	0.085

Note: The midazolam model was adopted from the compound library in Simcyp (Version 22). $P_{eff,man}$ human jejunum effective permeability; *Vss* volume of distribution at steady state; V_{max} maximum rate of metabolism; K_m Michaelis constant; *HLM* human liver microsome.

Table S2. Summary of drug-dependent parameters for the dabigatran etexilate PBPK model.

**Parameters**	**Dabigatran Etexilate**
**Physicochemical**	
Molecular weight (g/mol)	627.75
Log P	3.8
Compound type	Diprotic base
pKa	4.0, 6.7
Blood/plasma ratio	1.26
Fraction unbound	0.063
**Absorption**	
Absorption model	ADAM
$P_{eff,man}$ duodenum (10^{-4}cm/s)	0.113
$P_{eff,man}$ jejunum I (10^{-4}cm/s)	0.206
$P_{eff,man}$ jejunum II (10^{-4}cm/s)	0.144
$P_{eff,man}$ ileum I (10^{-4}cm/s)	0.058
$P_{eff,man}$ ileum II (10^{-4}cm/s)	0.058
$P_{eff,man}$ ileum III (10^{-4}cm/s)	0.057
$P_{eff,man}$ ileum IV (10^{-4}cm/s)	0.055
$P_{eff,man}$ colon (10^{-4}cm/s)	0.0001
**Distribution**	
Distribution model	Full PBPK Model
Vss (L/kg)	15.16
K_p scalar	1.0
**Elimination**	
Esterases-mediated pathways (HLS9)	
CES-1 V_{max} (pmol/min/mg)	19462
CES-1 K_m (μM)	33.5
fu_{inc}	0.69
Tissue scalar (liver:intestine:kidney)	1:0:0
CES-2 V_{max} (pmol/min/mg)	9050
CES-2 K_m (μM)	15.4
fu_{inc}	0.69
Tissue scalar (liver:intestine:kidney)	0.1:0:0
**Transport**	
Intestinal efflux	
P-gp J_{max} (pmol/min)	146
P-gp K_m (μM)	38.9
System	Caco-2
RAF/REF	0.99

Note: The dabigatran etexilate model was adopted from the literature [29].

$P_{eff,man}$ human jejunum effective permeability; *Vss* volume of distribution at steady state; K_p *scalar* scalar applied to all predicted tissue:plasma partition coefficients; *HLS9* human intestinal S9; *CES-1* Carboxylesterase 1; *CES-2* Carboxylesterase 2; V_{max} maximum rate of metabolism; K_m Michaelis constant; fu_{inc} fraction of unbound drug in the *in vitro* incubation; J_{max} *in vitro* maximum

rate of transporter-mediated efflux or uptake; *RAF/REF* relative activity factor or relative expression factor.

Table S3. Summary of drug-dependent parameters for the dabigatran and dabigatran glucuronide. PBPK models

**Parameters**	**Dabigatran**	**Dabigatran Glucuronide**
**Physicochemical**		
Molecular weight (g/mol)	471.52	647.65
Log P	-2.21	-4.15
Compound type	Ampholyte	Ampholyte
pKa	4.4, 12.4	4.14, 3.51
Blood/plasma ratio	0.69	0.89
Fraction unbound	0.65	0.225
**Distribution**		
Distribution model	Full PBPK Model	Minimal PBPK Model
Vss (L/kg)	0.96	0.16
K_p scalar	3.12	1.0
**Elimination**		
UGT-mediated pathways (recombinant)		
UGT2B15 V_{max} (µl/min per picomoles)	700	
UGT2B15 K_m (µM)	512	
rUGT scalars (liver:intestine:renal)	1:1:1	
Renal clearance (L/hour)	7.97	7.97
Additional systemic clearance (L/h)	0.97	0.97

Note: The dabigatran and dabigatran glucuronide models were adopted from the literature [29]. *Vss* volume of distribution at steady state; *K_p scalar* scalar applied to all predicted tissue:plasma partition coefficients; V_{max} maximum rate of metabolism; K_m Michaelis constant; *rUGT Scalars* tissue specific scalar for extrapolation of recombinant UGT in vitro kinetic data.

Table S4. Summary of drug-dependent parameters for the pitavastatin PBPK model.

**Parameters**	**Pitavastatin**
**Physicochemical**	
Molecular weight (g/mol)	421.46
Log P	2.91
Compound type	Monoprotic acid
pKa	5.31
Blood/plasma ratio	0.55
Fraction unbound	0.005
**Absorption**	
Absorption model	ADAM
$P_{eff,man}$ (10^{-4} cm/s)	4.688
Absorption scalar	1
**Distribution**	
Distribution model	Full PBPK
Vss (L/kg)	1.88
**Elimination**	
CYP-mediated pathways (recombinant)	
CYP2C8 CL_{int} (μL/min/pmol)	12.98
CYP2C9 CL_{int} (μL/min/pmol)	7.93
UGT-mediated pathways (recombinant)	
UGT1A3 CL_{int} (μL/min/pmol)	2.52
Additional clearance (HLM CL_{int}) (μL/min/mg protein)	1452.69
Renal clearance (L/hour)	0.129
**Transport**	
Hepatic uptake	
OATP1B1 $CL_{int,\,T}$ (μL/min/ million cells)	58.4
RAF/REF	14
OATP1B3 $CL_{int,\,T}$ (μL/min/ million cells)	5.1
RAF/REF	14

Note: The pitavastatin model was adopted from the literature [30], and the RAF/REF values for OATP1B1 and OATP1B3 were modified using pharmacogenomic data [31].

$P_{eff,man}$ human jejunum effective permeability; *Vss* volume of distribution at steady state; CL_{int} intrinsic clearance; *HLM* human liver microsome; $CL_{int,T}$ transporter-mediated intrinsic clearance; *RAF/REF* relative activity factor or relative expression factor.

Table S5. Summary of drug-dependent parameters for the atorvastatin PBPK model.

Parameters	Atorvastatin
**Physicochemical**	
Molecular weight (g/mol)	558.6
Log P	4.15
Compound type	Monoprotic acid
pKa	4.46
Blood/plasma ratio	0.61
Fraction unbound	0.023
**Absorption**	
Absorption model	ADAM
$P_{eff,man}$ (10^{-4} cm/s)	2.0875
Absorption scalar	1
**Distribution**	
Distribution model	Full PBPK
Vss (L/kg)	5.0648
**Elimination**	
CYP-mediated pathways (recombinant)	
CYP3A4 V_{max} (pmol/min/pmol) (ortho-hydroxylation)	43.95
CYP3A4 K_m (μM) (ortho-hydroxylation)	28.6
CYP3A4 V_{max} (pmol/min/pmol) (para-hydroxylation)	44.7
CYP3A4 K_m (μM) (para-hydroxylation)	24.6
CYP2C8 V_{max} (pmol/min/pmol) (para-hydroxylation)	0.12
CYP2C8 K_m (μM) (para-hydroxylation)	34.5
UGT-mediated pathways (recombinant)	
UGT1A1 V_{max} (pmol/min/mg protein)	2
UGT1A1 K_m (μM)	1.67
rUGT Scalars (liver:intestine:renal)	1:1:1
UGT1A3 V_{max} (pmol/min/mg protein)	38
UGT1A3 K_m (μM)	3.34
rUGT scalars (liver:intestine:renal)	0.25:0.25:0
UGT2B7 V_{max} (pmol/min/mg protein)	3.7
UGT2B7 K_m (μM)	16.72
rUGT scalars (liver:intestine:renal)	1:1:1
CLint (bile) (μL/min/10^6)	0.93
Renal clearance (L/hour)	6.58
**Transport**	
Intestinal uptake and efflux	
P-gp J_{max} (pmol/min)	141
P-gp K_m (μM)	115
System	Caco-2
RAF/REF	0.99
BCRP $CL_{int,T}$ (μL/min)	6
RAF/REF	1

Hepatic uptake	
NTCP J_{max} (μL/min/ million cells)	13616
NTCP K_m (μM)	185
OATP1B1 J_{max} (μL/min/ million cells)	745.51
OATP1B1 K_m (μM)	0.77
OATP1B3 J_{max} (μL/min/ million cells)	467.02
OATP1B3 K_m (μM)	0.73
OATP2B1 J_{max} (μL/min/ million cells)	449.92
OATP2B1 K_m (μM)	2.84
RAF/REF	1

Note: $CL_{int,T}$ for intestinal BCRP from the literature [26] was the only modification made to the built-in atorvastatin model in Simcyp (Version 22).

$P_{eff,man}$ human jejunum effective permeability; *Vss* volume of distribution at steady state; V_{max} maximum rate of metabolism; K_m Michaelis constant; *rUGT Scalars* tissue specific scalar for extrapolation of recombinant UGT in vitro kinetic data; J_{max} in vitro maximum rate of transporter-mediated efflux or uptake; $CL_{int,T}$ transporter-mediated intrinsic clearance; *RAF/REF* relative activity factor or relative expression factor.

Table S6. Summary of drug-dependent parameters for the rosuvastatin PBPK model.

**Parameters**	**Rosuvastatin**
**Physicochemical**	
Molecular weight (g/mol)	481.54
Log P	2.40
Compound type	Monoprotic acid
pKa	4.27
Blood/plasma ratio	0.625
Fraction unbound	0.107
**Absorption**	
Absorption model	ADAM
$P_{eff,man}$ (10^{-4} cm/s)	0.16105
Absorption scalar (Duodenum)	1
Absorption scalar (Jejunum I)	1
Absorption scalar (Jejunum II)	1
Absorption scalar (Ileum I)	1
Absorption scalar (Ileum II)	1
Absorption scalar (Ileum III)	1
Absorption scalar (Ileum IV)	1
Absorption scalar (Colon)	0.109
**Distribution**	
Distribution model	Full PBPK
Vss (L/kg)	0.11748
**Elimination**	
Additional clearance (HLM CL_{int}) (μL/min/mg protein)	3.224
Renal clearance (L/hour)	13.6
**Transport**	
Intestinal uptake and efflux	
OATP2B1 $CL_{int, T}$ (μL/min/ pmol transporter)	0.1
BCRP J_{max} (pmol/min/pmol Transporter)	2233
BCRP K_m (μM)	4.29
ISEF	1
Hepatic uptake and efflux	
NTCP $CL_{int, T}$ (μL/min/ million cells)	13.2
OATP1B1 $CL_{int, T}$ (μL/min/ million cells)	130
OATP1B3 $CL_{int, T}$ (μL/min/ million cells)	26.5
OATP2B1 $CL_{int, T}$ (μL/min/ million cells)	46.4
MRP4 $CL_{int, T}$ (μL/min/ million cells)	6.46
BCRP $CL_{int, T}$ (μL/min/ million cells)	6.46
RAF/REF	1
Renal uptake and efflux	
OAT3 $CL_{int, T}$ (μL/min/ million cells)	150
BCRP $CL_{int, T}$ (μL/min/ million cells)	150
RAF/REF	1

Note: $CL_{int,T}$ for renal OAT3 and BCRP from the literature [33] were the only modifications made to the built-in rosuvastatin model in Simcyp (Version 22).

$P_{eff,man}$ human jejunum effective permeability; *Vss* volume of distribution at steady state; *HLM* human liver microsome; $CL_{int,T}$ transporter-mediated intrinsic clearance; J_{max} in vitro maximum rate of transporter-mediated efflux or uptake; K_m Michaelis constant; *ISEF* activity abundance ratio between the intestine and in vitro cell system; *RAF/REF* relative activity factor or relative expression factor.

Table S7. Pharmacokinetics of five substrates in Chinese healthy volunteers and ESRD patients.

Compound	Population	Sample Size	AUC_{0-t} (ng·h /mL)	C_{max} (ng/mL)
Atorvastatin	HV	14	0.15±0.05	0.03±0.01
	ESRD	9	0.21±0.12	0.03±0.02
Dabigatran	HV	14	2.33±0.74	0.31±0.09
	ESRD	10	7.14±2.05	0.40±0.12
Midazolam	HV	14	0.13±0.06	0.06±0.03
	ESRD	10	0.04±0.02	0.02±0.01
Pitavastatin	HV	14	0.51±0.10	0.23±0.07
	ESRD	10	1.00±0.41	0.26±0.09
Rosuvastatin	HV	14	0.66±0.31	0.08±0.04
	ESRD	10	0.69±0.25	0.06±0.02

AUC_{0-t} area under the curve up to 24 hours; C_{max} maximum concentration; *HV* healthy volunteers; *ESRD* end-stage renal disease patients; data are given as mean±SD.

Table S8. Changes in unbound fraction in ESRD patients.

Drug	Increased Ratio[a]	f_u	
		Original[b]	Modified
Atorvastatin	1.36	0.023	0.031[c]
Midazolam	-	0.032	0.065[d]
Pitavastatin	1.67	0.005	0.008[c]
Rosuvastatin	1.25	0.107	0.134[c]

f_u unbound fraction.

[a]The increased ratio was calculated based on the observed ratio of ESRD to healthy volunteers reported in the literature [41].

[b]The original f_u values were the default parameters in drug models.

[c]The modified f_u values for statins in ESRD patients were calculated according to the increased ratio compared to healthy volunteers.

[d]Since the measured f_u in healthy volunteers was lower than the value reported in FDA Label (SEIZALAM)[41,43], the modified f_u adopted the value of patients with renal impairment as reported in the literature [42].

Table S9. Input K_i values for inhibitors and their metabolites from the Simcyp inhibitor library.

	Rifampicin	Itraconazole[a]	Clarithromycin	Ritonavir	Verapamil[b]
CYP3A4 K_i (μM)	15	0.0013/0.0023	10	0.00194	
CYP3A4 K_{app} (μM)			12	0.18	2.21/10.3
CYP3A4 K_{inact} (1/h)			2.13	19.8	2/6
CYP3A5 K_i (μM)				0.00194	
CYP3A5 K_{app} (μM)				0.18	3.99/4.53
CYP3A5 K_{inact} (1/h)				19.8	1.84/4.2
CYP2C8 K_i (μM)	24.5				
CYP2C8 K_{app} (μM)					
CYP2C8 K_{inact} (1/h)					
CYP2C9 K_i (μM)					
CYP2D6 K_i (μM)				0.04	
UGT1A3 K_i (μM)					
NTCP K_i (μM)	187.65	-/5.15			
OATP1B1 K_i (μM)	0.162	-/0.225	0.35		
OATP1B3 K_i (μM)	0.088	0.71/0.099	0.7		
OATP2B1 K_i (μM)	0.023	2.43/0.68			
MRP4 K_i (μM)	87.42				
P-gp K_i (μM)	4.3	0.00939/0.096	4	0.03	0.16/0.04
BCRP K_i (μM)	12.54	1.04/0.24	411		
OAT3 K_i (μM)					

K_i inhibition constant; K_{app} concentration for mechanism-based inhibitor; K_{inact} inactivation rate of enzyme.

[a]Itraconazole and OH-itraconazole.

[b]Verapamil and norverapamil.

Table S10. Summary of drug-dependent parameters for the valsartan PBPK model.

Parameters	Valsartan
**Physicochemical**	
Molecular weight (g/mol)	435.52
Log P	3.49
Compound type	Diprotic acid
pKa	4.73, 3.90
Blood/plasma ratio	0.56
Fraction unbound	0.056
**Absorption**	
Absorption model	ADAM
$P_{eff,man}$ (10^{-4} cm/s)	0.62196
Absorption scalar	1
**Distribution**	
Distribution model	Full PBPK
Vss (L/kg)	0.098
**Elimination**	
CYP-mediated pathways (HLM)	
CYP2C9 V_{max} (pmol/min/mg protein)	112.9
CYP2C9 K_m (μM)	46.73
fu_{mic}	0.885
Renal clearance (L/hour)	0.62
**Transport**	
Intestinal efflux	
MRP2 J_{max} (pmol/min)	268.5
MRP2 K_m (μM)	30.4
RAF/REF	1.67
Hepatic uptake	
OATP1B1 $CL_{int, T}$ (μL/min/ million cells)	0.624
RAF/REF	5.202
OATP1B3 $CL_{int, T}$ (μL/min/ million cells)	0.384
RAF/REF	4.3
MRP2 J_{max} (pmol/min)	447.5
MRP2 K_m (μM)	30.4
RAF/REF	0.37

Note: The valsartan model was adopted from the compound library in Simcyp (Version 22).

$P_{eff,man}$ human jejunum effective permeability; *Vss* volume of distribution at steady state; V_{max} maximum rate of metabolism; K_m Michaelis constant; fu_{mic} fraction of unbound drug in the in vitro microsomal incubation; J_{max} in vitro maximum rate of transporter mediated efflux or uptake; *RAF/REF* relative activity factor or relative expression factor; $CL_{int,T}$ transporter-mediated intrinsic clearance.

Table S11. Summary of dose-normalized predicted PK parameters in healthy volunteers.

Compound	Race	Age (years)	Female (%)	Sample Size	Dose, Route	AUC/Dose (ng·h /mL/mg)			C_{max}/Dose (ng/mL/mg)		
						Pre	Obs	P/O Ratio	Pre	Obs	P/O Ratio
Atorvastatin	Caucasian [40]	19-55	NA	12	0.1 mg, p.o.	1.40	1.19	1.18	0.30	0.14	2.16
	Caucasian [41]	50-71	50	6	0.1 mg, p.o.	1.66	2.92	0.57	0.39	0.22	1.78
	Chinese [27]	21-59	64.3	14	0.1 mg, p.o.	2.43	1.48	1.64	0.55	0.27	2.05
	Chinese [50]	20-30	0	45	20 mg, p.o.	2.70	2.00	1.35	0.55	0.54	1.03
	Chinese [52]	22±1	NA	18	20 mg, p.o.	2.37	2.74	0.86	0.51	0.43	1.19
	Chinese [51]	24.8±2.9	0	16	40 mg, p.o.	2.78	2.02	1.38	0.57	0.51	1.13
Pitavastatin	Caucasian [40]	19-55	NA	12	0.01 mg, p.o.	23.30	46.10	0.51	13.00	19.10	0.68
	Caucasian [41]	50-71	50	6	0.01 mg, p.o.	27.00	52.00	0.52	14.00	16.40	0.85
	Caucasian [49]	NA	0	18	2 mg, i.v.	47.69	41.95	1.14	31.31	29.92	1.05
	Caucasian [49]	NA	0	18	2 mg, p.o.	22.05	21.48	1.03	13.24	9.29	1.42
	Caucasian [49]	NA	50	24	4 mg, p.o.	23.35	42.88	0.54	12.96	19.06	0.68
	Chinese [27]	21-59	64.3	14	0.01 mg, p.o.	38.00	51.10	0.74	18.00	23.32	0.77
Rosuvastatin	Caucasian [40]	19-55	NA	12	0.05 mg, p.o.	3.60	5.14	0.70	0.40	0.54	0.74
	Caucasian [41]	50-71	50	6	0.05 mg, p.o.	3.80	5.84	0.65	0.49	0.43	1.15

	Chinese [27]	21-59	64.3	14	0.05 mg, p.o.	7.60	13.24	0.57	1.00	1.67	0.60
	Chinese [53]	18-24	0	10	10 mg, p.o.	8.41	9.55	0.88	1.02	0.81	1.25
	Chinese [54]	21-41	33	12	10 mg, p.o.	9.63	11.30	0.85	1.07	1.26	0.85
Dabigatran	Caucasian [40]	19-55	NA	12	0.375 mg, p.o.	5.01	3.84	1.31	0.51	0.46	1.10
	Caucasian [41]	50-71	50	6	0.375 mg, p.o.	5.73	3.09	1.85	0.53	0.35	1.50
	Chinese [27]	21-59	64.3	14	0.375 mg, p.o.	6.69	6.20	1.08	0.69	0.84	0.83
Midazolam	Caucasian [40]	19-55	NA	12	0.01 mg, p.o.	11.00	19.70	0.56	4.00	5.57	0.72
	Caucasian [41]	50-71	50	6	0.01 mg, p.o.	13.00	27.30	0.48	4.00	7.32	0.55
	Chinese [27]	21-59	64.3	14	0.01 mg, p.o.	19.00	13.49	1.41	5.00	5.69	0.88

AUC area under the curve; C_{max} maximum concentration; *Pre* predicted; *Obs* observed; *NA* not applicable; *p.o.* per os; *iv* intravenous; *P/O Ratio* was calculated as Pre/Obs.

Table S12. Simulated drug-drug interactions in Caucasian healthy volunteers.

Substrate Dosing Regimen	Perpetrator Dosing Regimen	$AUCR_{w/w-o}$		
		Pre	Obs	P/O Ratio
Atorvastatin 0.1mg, p.o.	Rifampin 600mg SD, p.o.	6.93	8.57	0.81
	Itraconazole 200mg QD for 5 days, p.o.	3.71	5.58	0.67
	Clarithromycin 500mg BID for 5 days, p.o.	4.64	3.45	1.35
Pitavastatin 0.01mg, p.o.	Rifampin 600mg SD, p.o.	2.77	4.23	0.65
	Itraconazole 200mg QD for 5 days, p.o.	1.16	0.96	1.21
	Clarithromycin 500mg BID for 5 days, p.o.	2.58	1.24	2.09
Rosuvastatin 0.05mg, p.o.	Rifampin 600mg SD, p.o.	3.09	4.59	0.67
	Itraconazole 200mg QD for 5 days, p.o.	1.27	1.78	0.71
	Clarithromycin 500mg BID for 5 days, p.o.	1.49	1.56	0.96
Dabigatran etexilate 0.375mg, p.o.	Rifampin 600mg SD, p.o.	1.39	2.38	0.58
	Itraconazole 200mg QD for 5 days, p.o.	1.45	7.41	0.20
	Clarithromycin 500mg BID for 5 days, p.o.	1.34	4.22	0.32
Midazolam 0.01mg, p.o.	Rifampin 600mg SD, p.o.	1.27	0.94	1.35
	Itraconazole 200mg QD for 5 days, p.o.	11.91	7.06	1.69
	Clarithromycin 500mg BID for 5 days, p.o.	6.36	4.85	1.31

[a] Data from a drug–drug interaction study involving microdose substrates [40].

$AUCR_{w/w-o}$ area under the curve ratio (with/without inhibitor); *Pre* predicted; *Obs* observed; *p.o.* per os; *SD* single-dose; *QD* once a day; *BID* twice a day. *P/O Ratio* was calculated as Pre/Obs.

Table S13. The predicted PK parameters for *SLCO1B*1 polymorphism phenotype in healthy volunteers.

Compound	Race	Dose, Route	Sample Size	*SLCO1B1* Phenotype	AUC (ng/mL·h)			C_{max} (ng/mL)		
					Pre	Obs	P/O Ratio	Pre	Obs	P/O Ratio
Atorvastatin	Caucasian [34]	20 mg, p.o.	16	ET	30.49	24.20	1.26	7.75	5.70	1.36
			12	IT	37.65	36.20	1.04	9.37	8.03	1.17
			4	PT	44.84	59.30	0.76	10.87	10.06	1.08
	Caucasian [35]	40 mg, p.o.	24	ET	54.87	68.40	0.80	13.63	13.20	1.03
			5	IT	68.48	111.50	0.61	16.71	18.30	0.91
	Chinese [35]	40 mg, p.o.	26	ET	102.20	104.30	0.98	20.13	20.30	0.99
			4	IT	111.85	188.40	0.59	21.82	35.40	0.62
Pitavastatin	Caucasian [31]	2 mg, p.o.	11	ET	43.80	81.10	0.54	27.71	31.20	0.89
			3	PT	74.79	250.00	0.30	41.68	129.00	0.32
Rosuvastatin	Caucasian [34]	10 mg, p.o.	16	ET	50.28	35.00	1.44	6.71	4.21	1.59
			12	IT	54.80	55.00	1.00	8.15	6.38	1.28
			4	PT	62.86	56.70	1.11	9.54	7.53	1.27
	Caucasian [35]	20 mg, p.o.	24	ET	76.66	90.70	0.85	10.18	8.20	1.24
			5	IT	86.76	124.10	0.70	11.84	11.50	1.03
	Chinese [35]	20 mg, p.o.	26	ET	165.18	167.40	0.99	17.35	17.40	1.00
			4	IT	168.23	227.70	0.74	17.99	24.90	0.72

AUC area under the curve; C_{max} maximum concentration; *p.o.* per os; *Pre* predicted; *Obs* observed; *P/O Ratio* was calculated as Pre/Obs; *ET* extensive transporter; *IT* intermediate transporter; *PT* poor transporter.

Table S14. The predicted PK parameters for *ABCG2* polymorphism phenotypes in healthy volunteers.

Compound	Race	Dose, Route	Sample Size	*ABCG2* Phenotype	AUC (ng/mL·h)			C_{max} (ng/mL)		
					Pre	Obs	P/O Ratio	Pre	Obs	P/O Ratio
Atorvastatin	Caucasian [35]	40 mg, p.o.	24	ET	59.85	72.10	0.83	14.73	13.30	1.11
			5	IT	56.94	86.90	0.66	14.24	17.30	0.82
	Chinese [35]	40 mg, p.o.	12	ET	95.99	121.90	0.79	18.61	25.80	0.72
			15	IT	100.05	99.40	1.01	19.97	17.40	1.15
			2	PT	100.92	212.40	0.48	20.67	42.80	0.48
Rosuvastatin	Caucasian [36]	10 mg, p.o.	14	ET	33.97	27.49	1.24	4.59	3.40	1.35
			2	IT	39.03	26.85	1.45	5.96	2.00	2.98
	Caucasian [35]	20 mg, p.o.	24	ET	71.80	88.80	0.81	9.66	7.90	1.22
			5	IT	88.29	138.00	0.64	12.60	13.50	0.93
	Chinese [35]	20 mg, p.o.	12	ET	120.52	140.90	0.86	11.91	15.20	0.78
			15	IT	173.25	180.70	0.96	18.54	18.40	1.01
			2	PT	242.04	447.40	0.54	30.23	50.00	0.60

AUC area under the curve; C_{max} maximum concentration; *p.o.* per os; *Pre* predicted; *Obs* observed; *P/O Ratio* was calculated as Pre/Obs; *ET* extensive transporter; *IT* intermediate transporter; *PT* poor transporter.

Table S15. Physiological parameters of HVs and ESRD patients.

Physiological Parameter	HV	ESRD
Age (years) (range) [a]	23-55	20-86
Height (cm) (CV%) [a]	M: 168.47 (2.59)	M: 166.80 (3.12)
	F: 155.75 (2.42)	F: 154.98 (3.52)
Weight (kg) (CV%) [a]	M: 67.00 (14.86)	M: 63.87 (16.96)
	F: 50.00 (8.83)	F: 54.23 (18.08)
Serum creatinine (umol/l) (CV%) [a]	M: 151.73 (139.50)	M: 787.74 (45.41)
	F: 76.25 (32.49)	F: 634.12 (37.96)
Hematocrit (%) (CV%)	M: 45.3 (9.5) [b]	M: 33.1 (17.2) [c]
	F: 40.5 (10.9) [b]	F: 32.7 (16.5) [c]
AGP (g/L) (CV%)	M: 0.683 (23.00) [b]	M: 1.0388 (39.00) [c]
	F: 0.616 (24.00) [b]	F: 1.0948 (34.08) [c]
HSA (g/L) (CV%)	M: 49.459 (7.3) [b]	M: 35.12 (27.38) [c]
	F: 47.249 (7.2) [b]	F: 34.43 (26.75) [c]

M male; *F* female; *AGP* α-acid glycoprotein; *HSA* human serum albumin.

[a] Data were obtained from the China Kidney Disease Network (CK－NET) 2016 annual data report [44].

[b] Data were collected from the built-in parameters of "Sim-Chinese Healthy Volunteers" model in Simcyp (Version 22).

[c] Data were collected from outpatient ESRD patients at Peking University Third Hospital (between 2013 and 2023).

Table S16. Simulation of muscle exposure in healthy volunteers and ESRD patients.

Substrate	Substrate Dosing Regimen	Simulated Muscle AUC (ng/mL)		
		HV	ESRD	ESRD/HV
Atorvastatin	20 mg SD	130.13	268.2	2.06
	20 mg QD, 8-14 days	143.81	442.47	3.08
Pitavastatin	2 mg SD	2.75	4.3	1.56
	2 mg QD, 8-14 days	2.88	5.98	2.08
Rosuvastatin	10 mg SD	5.20	10.16	1.95
	10 mg QD, 8-14 days	6.45	14.93	2.31

AUC area under the curve; *HV* healthy volunteers; *ESRD* end-stage renal disease patients; *SD* single-dose; *QD* once a day.

Table S17. Simulation of drug–drug interactions in healthy volunteers and ESRD patients.

Substrate	Substrate Dosing Regimen	Perpetrator Dosing Regimen	Simulated $AUCR_{w/w\text{-}o}$			Simulated $C_{max}R_{w/w\text{-}o}$		
			HV	ESRD	ESRD/HV	HV	ESRD	ESRD/HV
Atorvastatin	20 mg SD	Rifampin 600 mg SD	6.87	4.47	0.65	6.29	4.30	0.68
	20 mg QD, 8-14 days	Ritonavir 100 mg BID, 14 days	6.63	4.85	0.73	2.45	2.94	1.20
		Verapamil 80 mg TID, 14 days	2.05	1.92	0.94	1.56	1.73	1.11
		Itraconazole 200 mg QD, 14 days	7.14	8.97	1.26	3.03	5.95	1.96
		Clarithromycin 250 mg BID, 14 days	3.47	6.36	1.83	2.82	4.56	1.62
Pitavastatin	2 mg SD	Rifampin 600 mg SD	3.86	2.51	0.65	2.21	1.66	0.75
	2 mg QD, 8-14 days	Itraconazole 200 mg QD, 14 days	1.41	1.99	1.41	1.22	1.43	1.17
		Clarithromycin 250 mg BID, 14 days	2.25	3.98	1.77	1.75	2.07	1.18
Rosuvastatin	10 mg SD	Rifampin 600 mg SD	3.07	4.29	1.40	5.34	5.36	1.00
	10 mg QD, 8-14 days	Itraconazole 200 mg QD, 14 days	1.41	2.11	1.50	1.53	2.45	1.60
		Clarithromycin 250 mg BID, 14 days	1.30	1.31	1.01	1.44	1.34	0.93
Valsartan	80 mg SD	Rifampin 600 mg SD	1.77	1.71	0.97	1.48	1.86	1.26
	80 mg QD, 8-14 days	Itraconazole 200 mg QD, 14 days	1.26	1.85	1.47	1.15	1.72	1.50
		Clarithromycin 250 mg BID, 14days	1.34	2.72	2.03	1.24	2.45	1.98

$AUCR_{w/w\text{-}o}$ area under the curve ratio with/without perpetrator; $C_{max}R_{w/w\text{-}o}$ maximum concentration ratio with/without perpetrator; *HV* healthy volunteers; *ESRD* end-stage renal disease patients; *SD* single-dose; *QD* once a day; *BID* twice a day; *TID* three times a day
